# Controlled release of MIF siRNA and GDNF protein from a photocurable scaffold efficiently repairs spinal cord injury

**DOI:** 10.1002/mco2.70099

**Published:** 2025-02-17

**Authors:** Yan Gao, Kaiyu Wang, Yi Wu, Shan Wu, Pingchuan Ma, Jin Zhang, Jingmei Li, Guobo Shen, Ke Men

**Affiliations:** ^1^ Department of Biotherapy Cancer Center and State Key Laboratory of Biotherapy West China Hospital Sichuan University Chengdu P.R. China; ^2^ State Key Laboratory of Oral Diseases & National Center for Stomatology & National Clinical Research Center for Oral Diseases & Department of Head and Neck Oncology West China Hospital of Stomatology Sichuan University Chengdu P.R. China

**Keywords:** controlled‐release, hydrogel, nanoparticle, siRNA‐based gene therapy, spinal cord injury

## Abstract

Compared with traditional treatment strategies, siRNA‐based gene therapy combines with protein therapy to offer a new strategy for spinal cord injury (SCI). The siRNA and protein therapy are limited by the large and deep lesion site and local co‐delivery vectors. However, the photocurable scaffold has the properties of injectable, flexible, and biodegradable, which provide a potential formulation for siRNA and protein combined therapy. Here, a photocurable lipid nanoparticle gel (PLNG) scaffold is designed for efficiently sustained and controlled release of the macrophage migration‐inhibitory factor (MIF) targeted siRNA and co‐delivery of GDNF protein for SCI. The GDNF is chemically modified in the scaffold and the prepared GDNF‐PLNG/siRNA scaffold is injectable with easily photocured. This formulation can inhibit inflammation by promoting macrophage M2 polarization and effectively promote primary neuron axon growth. After locally administered with GDNF‐PLNG/siMIF scaffold to SCI mice, the scaffold promoted neuron regeneration by upregulation of neuron cytokine production and inhibited inflammation through the downregulation immune pathway. With the interaction mechanism of GDNF and MIF siRNA, GDNF‐PLNG/siMIF scaffold increases the collagen and integrin expression to promote spinal cord repairing and significantly improve motor function, so that scaffold is a potential candidate gene formulation applied to clinical SCI treatment.

## Graphical abstract

We developed an injectable and photocurable controlled‐release scaffold PLNG for MIF siRNA and co‐delivery of GDNF protein at the SCI wound site. After treatment with only three doses, GDNF‐PLNG/siMIF scaffold successfully regulated the activities of neurons, microglia, and macrophages, thus promoting neuron axon regeneration, re‐myelination, and inhibiting neuroinflammatory.

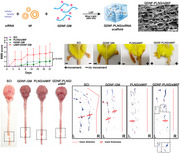

## 1 INTRODUCTION

Spinal cord injury (SCI) generates serious disabilities, including the loss of sensory and motor functions and other organs and loss of autonomic control.^1^, ^2^ The complicated injury process greatly impedes posttraumatic neuron regeneration and axon growth and complicates SCI therapy. However, it is hard to satisfy neuron regeneration and inhibit neuroinflammation at the same time. Thus, it is crucial to devise novel treatment approaches that integrate nervous system interventions with addressing inflammation for effective SCI therapy.

Gene therapy, leveraging its capability to introduce genetic materials into patients for the purpose of modulating disease‐specific genes, holds vast potential for development and significant application value within the realm of medicine.^3^, ^4^, ^5^ Based on the properties for inducing the silencing of target genes and inhibiting the expression of proteins, siRNA (small interfering RNA) gene therapy is an innovative approach to gene therapy that has made significant progress in medical research and clinical application.^6^, ^7^, ^8^ At present, siRNA can target genes that inhibit axon regeneration or active neuroinflammatory, such as CC chemokine ligand 2 (CCL2) and PTEN (phosphatase and tensin homolog).^9^, ^10^ During the secondary damage stages of SCI, the inflammatory response is active and the abundance of immune cells accumulates.^11^ These complicated immune reaction processes of SCI have offered potential therapeutic targets for siRNA‐based gene therapy. However, the siRNA nanoparticles complex is likely to be cleared from the body via hepatic and renal, increasing the frequency of dose. Moreover, the complicated environment of SCI requires that the siRNA genetic materials can be accurately delivered to target cells and should be highly concentrated at the target site with bioactive. Thus, it is essential to develop a new siRNA‐based gene therapeutic formulation for SCI.

Protein holds a vital importance in the life cycle and is widely applied in several diseases.^12^, ^13^, ^14^ Proteins could work as neurotrophic factors to support neuron growth, survival, and differentiation, such as glial cell line‐derived neurotrophic factor (GDNF), neurotrophin‐3 (NT‐3), and nerve growth factor (NGF).^15^, ^16^ At present, utilizing proteins such as nerve growth factor, anti‐inflammatory and antioxidant proteins, and tissue repair and regenerative proteins can facilitate the repair and regeneration of spinal cord injuries.^17^, ^18^, ^19^, ^20^ Neurons are highly differentiated cells and do not regenerate if damaged by external factors, while damaged axons could be regenerated with the nourishment of neurotrophic factors.^21^ The intricate mechanisms of SCI highlight the pivotal functions of associated neuronal cells, offering numerous potential therapeutic protein targets. However, the structure of protein is easily influenced by the microenvironment (temperature, pH) of SCI and easily destroyed by proteinase, causing poor stability by local injection administration and requiring a suitable vector to protect the bioactive in vivo. Hence, to accomplish effective combined gene and protein therapy for SCI, the development of a novel therapeutic formulation specifically tailored for SCI is imperative. Despite the siRNA target and protein therapy targeted could be choices, there is an urgent need for a carrier that can load both of them at the same time for SCI therapy. As the complicated pathology process and deep wound of SCI, drugs are required not to clear and prolong the time of drug effect. Moreover, the open wound in the spinal cord is prone to drug flow due to the movement of the spine. Thus, there is a need for an operational and controlled‐release drug delivery formulation for SCI.

Sustained and controlled release technology effectively enhances the safety of treatment and patient compliance by regulating the release rate of drugs, extending the duration of drug action, and reducing the frequency of drug administration.^22^, ^23^, ^24^ Among the various controlled‐release delivery carriers, scaffolds can control drug release by adjusting the molecular structure and physical properties of the materials. Furthermore, some environmentally responsive scaffolds are capable of rapidly completing the sol‐gel transition, which is beneficial for local treatment of SCI.^25^, ^26^, ^27^, ^28^, ^29^ However, there are more requirements for the delivery of bioactive medicine, including protection capacity, maintaining bioactive drug, injectable, controlled‐release, phase transition in vivo, and drug loading capacity. In this study, a photocurable lipid nanoparticle gel (PLNG) scaffold is introduced for efficiently sustained and controlled release of the MIF‐targeted siRNA and co‐delivery of GDNF protein for SCI treatment. The prepared GDNF‐PLNG/siRNA scaffold can be injected into the wound site and easily photocured with sol‐gel transformation. This scaffold load with siRNA targeted the macrophage migration‐inhibitory factor (MIF) gene and co‐delivery of GDNF protein, which is chemically modified on the backbone of the scaffold. Compared with the previously published article focused on treating SCI by co‐delivery dual siRNA, this article treats SCI by the co‐delivery siRNA and protein, in which the protein focuses on neurorepair and the siRNA is targeted at neuroinflammation.^30^ With the interaction mechanism of GDNF and MIF siRNA, GDNF‐PLNG/siMIF scaffold increases the growth factors to re‐constitution neuron signaling and causes a strong immune response to regulate the anti‐inflammatory pathway. Therefore, the tissue repairing and locomotor‐improving properties of the GDNF‐PLNG/siMIF scaffold proved that is a promising biomaterial formulation for SCI treatment.

## 2 RESULTS

### 2.1 Preparation and characterization of PLNG scaffold

**FIGURE 1:**
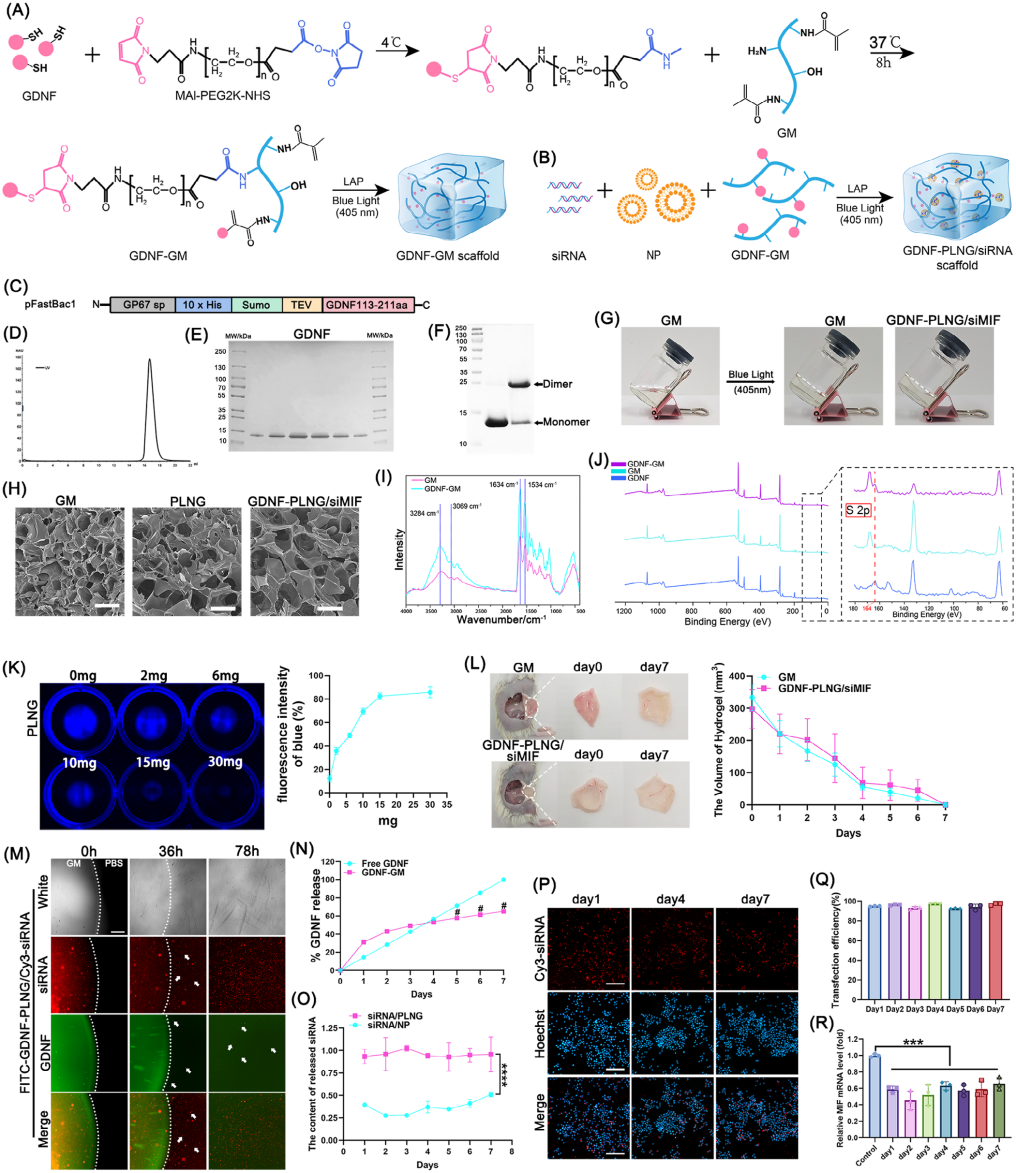
Preparation and characterization of GDNF‐PLNG/siRNA scaffold. (A) GDNF protein chemical modified to GM scaffold by MAL‐PEG2K‐NHS. (B) siRNA, NP, and GDNF‐GM mixed to obtain GDNF‐PLNG/siRNA scaffold. (C) schematic representation of pFastBac1‐GDNF. (D) Superdex200 HR gel‐filtration elution profiles of the purified GDNF. (E) the purified GDNF detected by SDS‐PAGE assay. The gel was stained with Coomassie Blue: Lane 1 was marker; Lanes 2–7 were samples of peak fractions containing the GDNF protein. (F) SDS‐PAGE analysis of the pooled GDNF: Lane 1, marker; Lane 2, GDNF protein sample was reduced by DTT; Lane 3, GDNF protein sample without DTT. (G) the image of prepolymer GM solution, GM scaffold, and GDNF‐PLNG/siMIF scaffold. (H) the microstructure of GM, PLNG, and GDNF‐PLNG/siMIF scaffold imaged by SEM (scale bar, 250 µm). (I) FT‐IR analysis of GM and GDNF‐GM scaffold. (J) XPS analysis of GDNF protein, GM scaffold, and GDNF‐GM scaffold. (K) the in vitro degradation and fluorescence intensity of PLNG scaffold stained with blue Alkene coupling hydrogel fluorescent dye. (L) the in vivo degradation and volume of GM and GDNF‐PLNG/siMIF scaffold during 7 days. (M) the release and degradation process of FITC‐GDNF‐PLNG/Cy3‐siRNA scaffold (scale bar, 400 µm). (N) the release rate of GDNF from the GM scaffold for seven days. (O) the amount of siRNA released from the PLNG scaffold over a period of seven days. (P) the supernatant of seven days transfected to RAW264.7 cells, and the nuclei stained with Hoechst (blue) for 15 min (scale bar, 100 µm). (Q) The transfection efficiency was evaluated using the supernatant from the 7 days. (R) The relative expression level of MIF mRNA was measured after treatment with the supernatant from the 7 days.

In this research, the ‐HS bond in GDNF was modified with maleimide (Mal) by Michael reaction, and then the ‐N‐hydroxysuccinimide (NHS) bond was modified with amine (NH_2_) in GM scaffold. Thus, the GM scaffold was chemically modified with GDNF protein and added visible‐light sensitive initiator LAP to obtain the GDNF‐GM scaffold (Figure 1A). In addition, the siRNA, NP, and GDNF‐GM were mixed and added with LAP to obtain the GDNF‐PLNG/siRNA scaffold (Figure 1B). The prepared GDNF‐PLNG/siRNA scaffold can be easily photocured with sol‐gel transformation when exposed to 405 nm blue light illumination.

To effectively deliver siRNA, NP was successfully prepared following our previous work. The mean particle size of NP measured 51.51 ± 3.2 nm and had a zeta potential value of 41.97 ± 1.2 mV (Figure S1A). The NP exhibited a spherical morphology and uniform size which was consistent with the measured particle size (Figure S1B). In addition, compared with PEI25K, the cell viability of NP was higher in 293T and RAW264.7 cells (Figure S1C). These results suggested that NP was positively charged and uniformly distributed nanoparticles with great cell safety. Moreover, NP could completely bind with siRNA at the ratio of 4:1, revealing that negatively charged siRNA could be encapsulated by NP to form a complex and this ratio was used for subsequent experiments (Figure S1D). After being encapsulated by NP, the particle size of the siRNA/NP complex increased slightly to 144.3 ± 11.55 nm and the zeta potential remained relatively unchanged at 49.46 ± 0.38 mV (Figure S1A). In addition, the morphology of the siRNA/NP complex remained spherical with an average diameter of 120 nm (Figure S1B). These findings indicated that while the particle size of the siRNA/NP complex increased, there was no discernible impact on its zeta potential or spherical shape. Furthermore, compared with siRNA treated with RNase, the siRNA/NP complex still detected the band on the gel even for a longer time, indicating that siRNA could be protected by NP to increase stability (Figure S1E). These results demonstrated that NP exhibited great biosafety and protected drugs from degradation.

To obtain substantial amounts of GDNF protein, a construct named pfastbac1‐GP67 sp‐His‐Sumo‐tev‐GDNF was successfully designed and produced (Figure 1C). We used a Sumo tag to improve the expression of the GDNF protein. The secreted GDNF fusion protein was successfully expressed in Sf9 cells using the Bac‐to‐Bac System. After large‐scale purification of the GDNF protein and the removal of the His‐Sumo tag, the mixture was further purified and subjected to SDS‐PAGE analysis. The product exhibited a monodispersed state in size‐exclusion chromatography (Figure 1D,E). The peak fractions were subsequently analyzed by SDS‐PAGE under reducing or nonreducing conditions, and the results indicated that the GDNF protein primarily exists in a dimeric form, consistent with its physiological activity as a dimer (Figure 1F).

Both in vitro and in vivo assessments were conducted to evaluate the physical characteristics and biodegradability of the GDNF‐PLNG/siMIF scaffold. Before being crosslinked under 405 nm blue light, the prepolymer GM solution was transparent and flowing liquid (Figure 1G). Once crosslinked, the GM scaffold was happened the change with sol to gel as the same as the GDNF‐PLNG/siMIF scaffold. These findings illustrated that the PLNG scaffold displayed photopolymerization capabilities with a swift sol‐to‐gel transition, and these properties could not be influenced by loading with siRNA and protein. The GM, PLNG, and GDNF‐PLNG/siMIF scaffold was subjected to freeze‐drying and imaged by SEM to observe the microstructure of hydrogel (Figure 1H). These could be observed in the internal honeycomb pore structure and three‐dimensional network structure in these scaffolds, explaining the drugs could be encapsulated in the porous and had the capability of carrying multiple drugs. Moreover, FT‐IR was detected to confirm the synthesis of GDNF‐GM scaffold (Figure 1I). The amide A band is at 3284 cm^−1^ and the amide B band was at 3069 cm^−1^, which was mainly caused by N‐H stretching vibration. Due to C = O stretching vibration, the amide I band was at 1634 cm^−1^. The amide II band absorption at 1534 cm^−1^ is mainly caused by the coupling of N–H bending vibration and C–N stretching vibration. Compared with the GM scaffold, the characteristic amide absorbance peak of the GDNF‐GM scaffold was similar to the GM scaffold, but the amide I band and the amide II band were significantly stronger than the corresponding peaks of the GM scaffold. These results were attributed to the newly formed amide bonds in the GDNF‐GM scaffold reaction and suggested that GDNF was successfully modified on the GM scaffold by MAL‐PEG_2000_‐NHS reaction. To confirm the chemical modification of the GDNF‐GM scaffold, the surface element content was compared by XPS (Figure 1J). The sulfur (S) element was the character of GDNF protein but not in GM hydrogel, in which the S 2p peak was positioned at 164 eV. Compared with the GM scaffold, the S 2p peak was observed in the GDNF and GDNF‐GM scaffold, suggesting that GDNF protein was successfully modified into the GM scaffold. To measure the fluidity of GM and GDNF‐PLNG/siMIF scaffold, the viscosity was detected (Figure S1F). As the shear rate increased, the viscosity of each scaffold decreased, proving that the GM and GDNF‐PLNG/siMIF scaffold was shear thinning and injectable. In order to detect whether the NP existed after scaffold degradation, the siMIF/PLNG scaffold was degraded by collagenase, and the supernatant was collected to examine the particle size and morphology (Figure S1G). The mean particle size and spherical morphology remained unchanged, suggesting that the nanoparticle properties were still maintained and without the influence of the scaffold. Furthermore, the biodegradation ability of the PLNG scaffold was evaluated. As shown in Figure 1K, the blue fluorescence of the PLNG scaffold was gradually turned small with the increase of collagenase, and the fluorescence intensity of the degraded PLNG scaffold gradually increased to 80%. In addition, the GM scaffold and GDNF‐PLNG/siMIF scaffold were subcutaneously injected into mice (Figure 1L). The scaffold was gradually degraded and completely disappeared on day 7 under the action of digestive enzymes in the body, showing great degradable and biosafety. In summary, these findings revealed that the PLNG scaffold exhibited modifiability and loaded multiple drugs with great degradable and biocompatibility.

### 2.2 The release function evaluated of GDNF‐PLNG/siRNA scaffold

To observe the GDNF and siRNA released from the PLNG scaffold, the FITC‐modified GDNF and Cy3‐siRNA were loaded into the PLNG scaffold. As shown in Figure 1M and Figure S2, the scaffold was completely degraded at 78 h and the drugs were also released, indicating that multiple drugs could be protected and released from the scaffold with controlled‐sustained release. In addition, the protein release rate from the scaffold was evaluated (Figure 1N). Compared with free GDNF, the release ratio of GDNF loaded in the scaffold was significantly slowed down on the fifth day, denoting that the scaffold could protect protein and improve its utilization via a controlled‐release delivery system. Moreover, the protection of siRNA in the scaffold was also evaluated (Figure 1O). The store of siRNA in the PLNG scaffold was more than 0.8 µg for seven days (the initial amount was 1 µg). Compared with siRNA loaded in the scaffold, almost half of siRNA encapsulated in NP was lost. These findings suggested that the scaffold exhibited the storage and protection ability of drugs for a long time and could release the drugs in a way of controlled‐sustained release. Meanwhile, the transfection ability and gene silencing ability of the released siRNA/NP complex from the scaffold were verified by transfecting to RAW264.7 cells. As depicted in Figure 1P,Q and Figure S3, the released siRNA/NP complex exhibited strong transfection fluorescence with no obvious cytotoxicity and the transfection efficiency of superannuant for 7 days. Furthermore, the released siRNA/NP complex also verified the gene silencing ability by transfecting macrophage cells. Compared with the control group, the relative expression MIF mRNA level in cells was decreased to 40% (Figure 1R), illustrating that siRNA/NP complex loaded in scaffold could still maintain the gene function in cells. In summary, the multiple drugs loaded in the PLNG scaffold could not only be released with controlled substances, but also the released drugs kept bioactive to work.

### 2.3 The anti‐inflammatory and axon regeneration mechanism of siMIF/NP complex and GDNF protein in vitro

**FIGURE 2:**
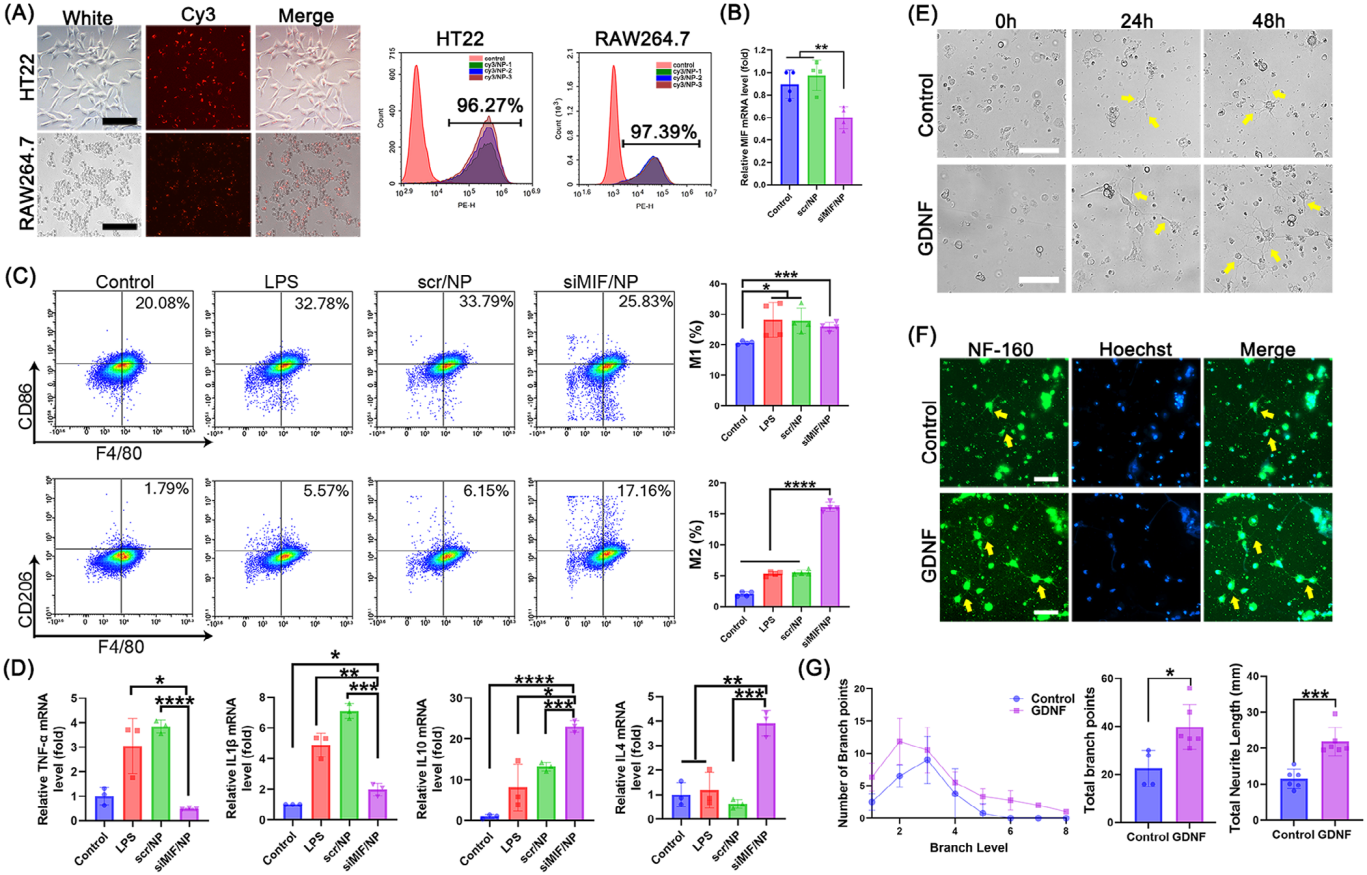
The function evaluation of MIF siRNA and GDNF protein in vitro. (A) The capability of the Cy3‐siRNA/NP complex to transfect HT22 and RAW264.7 cells was evaluated. (B) the gene silence ability of the MIF gene detected by QPCR. (C) the flow cytometer analysis of M1 (F4/80^+^ CD86^+^) and M2 (F4/80^+^ CD206^+^) treated by siMIF/NP complex. (D) the expression of inflammatory factor (TNF‐α, IL1β) and anti‐inflammatory factor (IL10, IL4) after treated by siMIF/NP complex. (E) the image of GDNF treated to primary spinal cord neurons on 0, 24, and 48 h (scale bar, 100 µm). (F) After 48 h of treatment with GDNF, primary spinal cord neurons were visualized through staining with NF‐160 (green) and Hoechst (blue), as shown in the image with a scale bar of 50 µm. (G) The number of branch points, the total count of branch points, and the total neurite length were quantified.

The function of MIF siRNA delivered by NP and GDNF protein was evaluated in vitro. First, the capability of NP to deliver genetic material was assessed through the transfection of RAW264.7 and HT22 cells (Figure 2A). There were strong Cy3‐positive cells in RAW264.7 and HT22 cells with a high transfection efficiency, resulting in siRNA being delivered by NP with high transfection ability and siRNA could easily delivered to neuron and macrophage cell lines. Then the knockdown efficiency of MIF siRNA was detected by transfected RAW264.7 cells (Figure 2B). Compared with the control and scr/NP groups, the siMIF/NP complex group exhibited a 50% reduction in relative MIF mRNA levels, demonstrating the effective delivery and gene‐silencing capability of MIF siRNA.

To assess the anti‐inflammatory of the MIF gene, the siMIF/NP complex was treated with LPS‐stimulation RAW264.7 cells for flow cytometer and QPCR analysis. As shown in Figure 2C, compared with other groups, the M1 phenotype of F4/80^+^ CD86^+^ positive cells in the siMIF/NP group was 25.83% and the M2 phenotype of F4/80^+^ CD206^+^ positive cells in the siMIF/NP group was 17.16%. These findings suggested that the siMIF/NP complex exhibited the ability to promote M2 macrophage polarization. In addition, the expression of inflammatory and anti‐inflammatory factors was also evaluated (Figure 2D). Compared with other groups, the relative expression of TNF‐α and IL1β mRNA levels in the siMIF/NP group was significantly decreased to 0.5‐fold and twofold, respectively. Moreover, the relative expression of IL10 and IL4 mRNA levels in the siMIF/NP group was highly increased to 22‐fold and 3.3‐fold, respectively. These findings showed MIF siRNA could inhibit inflammation by promoting macrophage anti‐inflammatory M2 phenotype polarization.

Furthermore, GDNF protein was treated in primary spinal cord neurons to assess the neuron improvement function. Compared with the control, the neuron axon treated with GDNF began to grow at 24 h and exhibited plenty of neuron axon growth at 48 h (Figure 2E, as the yellow arrow indicates). After the neuron cells were stained with neurofilament medium antibody, more neurons had grown axons than the control with only 1–2 neurons (Figure 2F). In addition, the assessment encompassed the quantification of branch points, inclusive of the total count, as well as the total neurite length (Figure 2G). The number of branch points, branch level, total branch points, and total neurite length were all higher in the treated group, being twofold, 1.3‐fold, twofold, and twofold, respectively, higher than the control group. These findings revealed that GDNF protein could effectively promote neuron axon growth and favor axonal regeneration of neurons.

### 2.4 GDNF‐PLNG/siRNA scaffold promotes locomotor recovery of SCI

**FIGURE 3:**
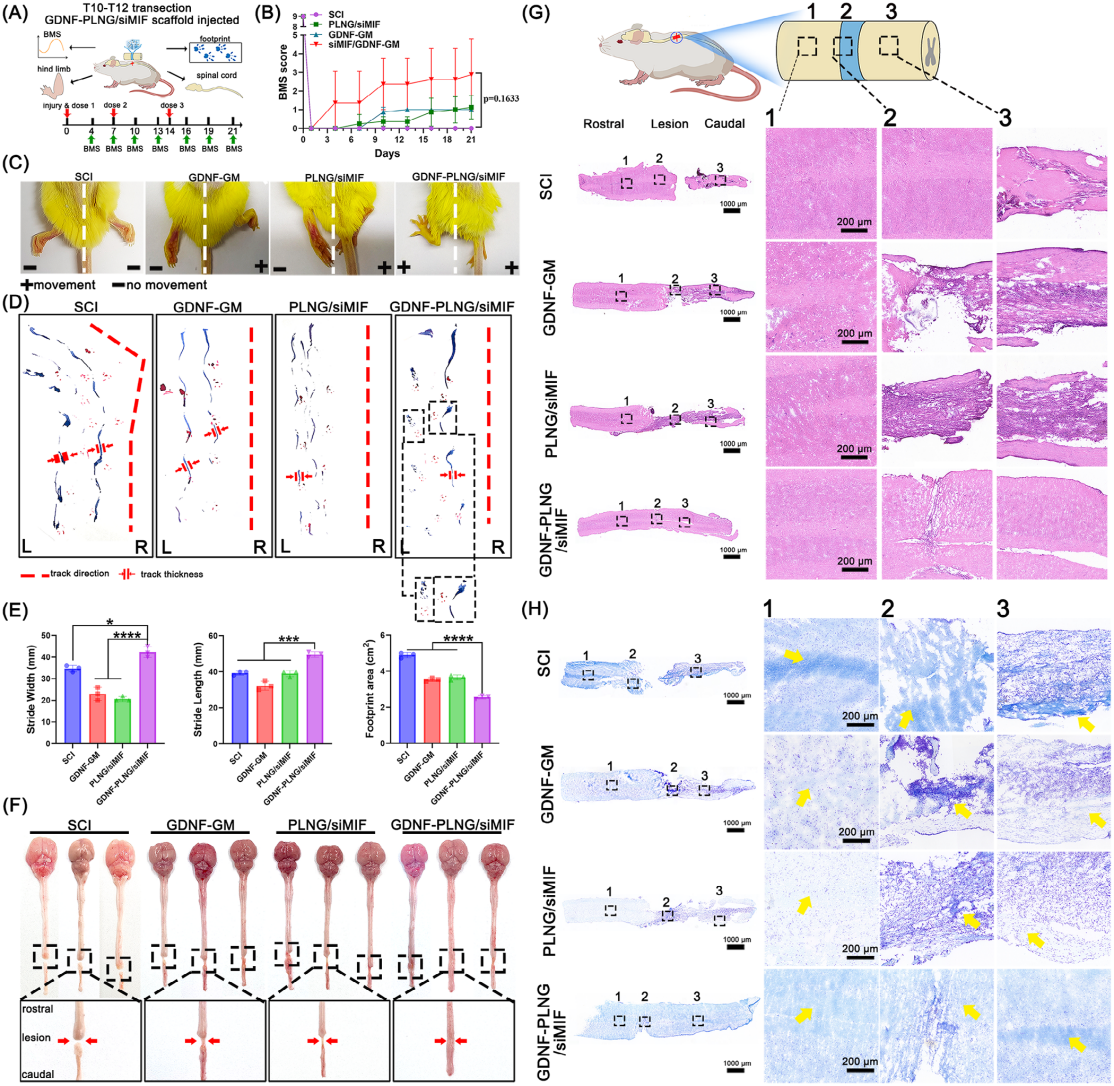
The therapy efficiency of GDNF‐PLNG/siMIF on SCI model. (A) the schematic diagram of treatment and locomotor evaluation. (B) The BMS score was collected every two days during therapy. (C) The image of the hind limb in each group after treatment. (D) The gait analysis of each group. red ink was regarded as forelimbs and the blue ink was regarded as hind limbs. (E) The statistical analysis of stride width, stride length, and footprint area based on the footprint. (F) The photograph of spinal cord tissue after treatment. (G) the overview of H&E staining in the spinal cord section (scale bar, 1000 µm). Alongside a magnified look at the rostral, lesion, and caudal (scale bar, 200 µm). (H) The overview of Luxol Fast Blue staining in spinal cord section (scale bar, 1000 µm). Alongside a magnified look at the rostral, lesion, and caudal (scale bar, 200 µm).

A mouse model of SCI was established to assess the therapeutic efficacy of the GDNF‐PLNG/siRNA scaffold through local administration. After being given three doses, the locomotor recovery of mouse was evaluated by the Basso mouse scale (BMS) score, hind limb, footprint, and spinal cord tissue (Figure 3A). First, the locomotor ability of the GDNF‐PLNG/siMIF group began to recover on day 4, while the other groups did not recover (Figure 3B). After treatment with three doses, the BMS score in the GDNF‐PLNG/siMIF group reached 3–4 points, which was higher than that of the GDNF‐GM and PLNG/siMIF groups. The SCI group without no significant recovery during the whole treatment. The findings suggest that administration of the GDNF‐PLNG/siMIF scaffold significantly enhances the BMS score and promotes locomotor recovery. As results shown in Figure 3C, both hind limbs of the SCI group were paralyzed with toe curling. Compared with other groups, the toe of the left hind limb in the GDNF‐PLNG/siMIF group was spread out strongly, accompanied by occasional plantar placement of the paw with weight support. Additionally, the right hind limb exhibited ankle joint movement. The findings denoted GDNF‐PLNG/siMIF scaffold effectively promoted the recovery of mouse toes and joints. In addition, the gait of each mouse was analyzed by staining with red or blue ink (Figure 3D). The track direction of the SCI group was curved lines with obvious track thickness. The GDNF‐GM and PLNG/siMIF groups exhibited straight track direction with slight track thickness. GDNF‐PLNG/siMIF group exhibited straight track direction and clear footprints with plantar stepping (as shown in a partially enlarged view). In contrast, the other groups exhibited sweeping movements without plantar stepping. Furthermore, compared with other groups, the stride width, stride length, and footprint characteristics in the GDNF‐PLNG/siMIF group were near normal levels (Figure 3E). The findings demonstrated that treatment with GDNF‐PLNG/siMIF significantly improved the locomotor stability of the mouse and promoted functional recovery.

Meanwhile, the spinal cord tissue of each group was collected and photographed to analyze tissue integrity (Figure 3F). Treatment with GDNF‐PLNG/siMIF scaffold showed a complete spinal cord structure without transection at the lesion site, illustrating that obviously promoted spinal cord repair and axon regeneration. In addition, the spinal cord was treated with H&E staining to observe the morphological changes (Figure 3G). The spinal cord tissue in the GDNF‐PLNG/siMIF group showed a connected and integrated tissue structure, favoring tissue regeneration and recombination the nerve conduction. To examine the pathological alterations in the nerve myelin sheath, the spinal cord underwent staining with Luxol Fast Blue (LFB), a copper‐based phthalocyanine dye (Figure 3H). Upon general inspection, it was evident that the myelin sheath in the SCI, GDNF‐GM, and PLNG/siMIF groups displayed signs of damage and incompleteness. However, when treated with the GDNF‐PLNG/siMIF scaffold, a distinct and intact morphology of the nerve myelin sheath was observed. The GDNF‐PLNG/siMIF group has more neuron cells in the lesion site and integrity myelinated fibers in the rostral and caudal sites. In summary, these results explained that treated with GDNF‐PLNG/siMIF scaffold could decrease tissue damage by promoting neuron and myelinated fibers and promote axon regeneration for combination nerve conduction.

### 2.5 The inflammatory response of GDNF‐PLNG/siRNA scaffold

**FIGURE 4:**
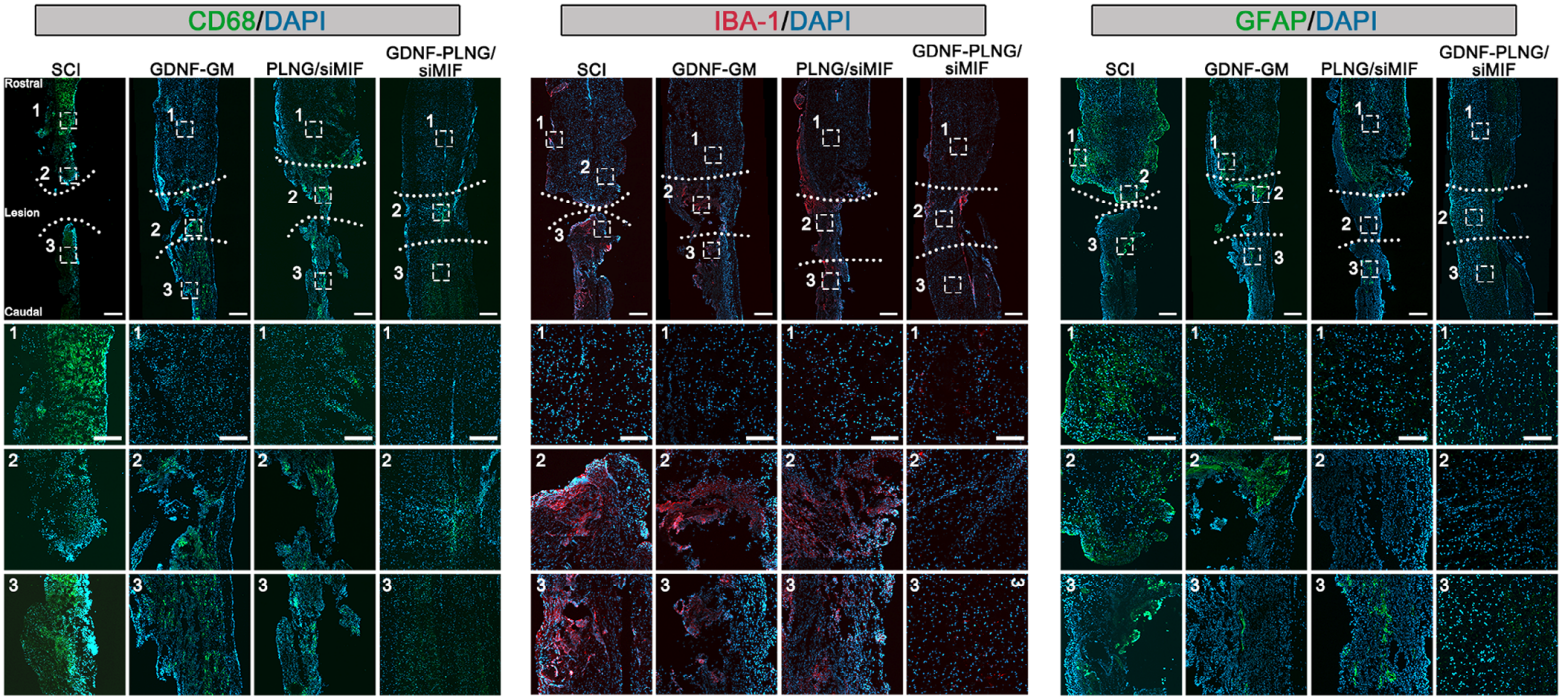
The immunofluorescence analysis of spinal cord sections involves the individual examination of CD68 (stained green), IBA1 (stained red), GFAP (stained green), and DAPI (stained blue), separately (scale bar, 500 µm). The regions of rostral, lesion, and caudal for each group undergo enlargement for inspection (scale bar, 200 µm).

To determine the effectiveness of the GDNF‐PLNG/siMIF scaffold on the inflammatory response after therapy, the expression level of CD68 (macrophage marker), IBA1 (microglia and macrophage marker), and GFAP (astrocyte marker) (Figure 4). There was less CD68 positive cell signal in the lesion site of the GDNF‐PLNG/siMIF group, while the rostral and caudal site of SCI had a strong CD68 positive cell signal. These results proved that GDNF‐PLNG/siMIF scaffold could decrease the proliferation of macrophages in the lesion site. In addition, compared with SCI, GDNF‐GM, and PLNG/siMIF groups, the number of IBA1 positive cells in the GDNF‐PLNG/siMIF group was decreased significantly, especially in the lesion site of the spinal cord. Moreover, there was a strong GFAP positive cells signal in the SCI group, the lesion site in GDNF‐GM group, and the caudal site in PLNG/siMIF group, while the number of GFAP positive cells in GDNF‐PLNG/siMIF group was decreased significantly, suggesting that GDNF‐PLNG/siMIF scaffold could inhibit astrocytes proliferation. In summary, treatment with GDNF‐PLNG/siMIF scaffold exhibited strong anti‐inflammatory ability and inhibited glial scar formation at the lesion site of spinal cord tissue.

### 2.6 The neuron recovery of GDNF‐PLNG/siRNA scaffold

**FIGURE 5:**
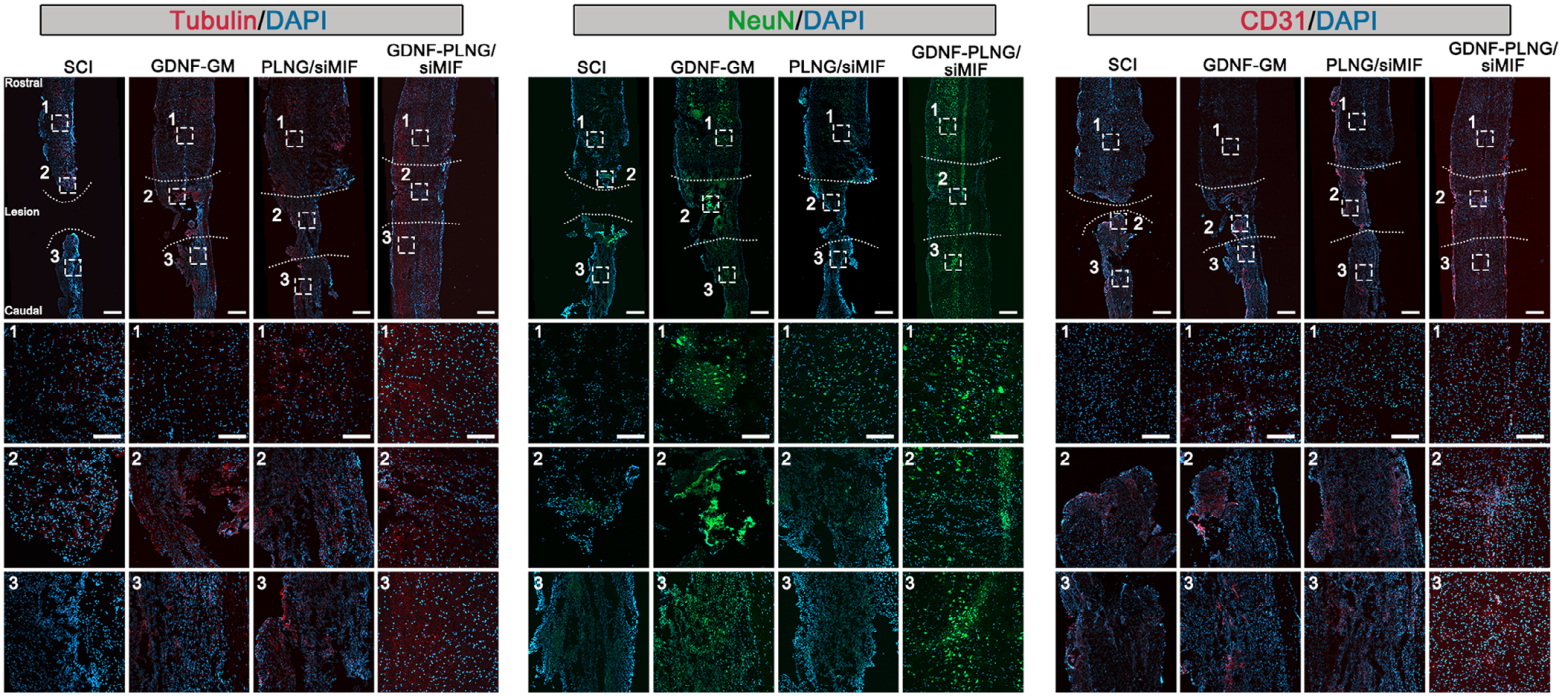
The immunofluorescence analysis of spinal cord sections involves the individual examination of NeuN (stained green), CD31 (stained red), Tubulin (stained red), and DAPI (stained blue), separately (scale bar, 500 µm). The regions of rostral, lesion, and caudal for each group undergo enlargement for inspection (scale bar, 200 µm).

To determine the effect of GDNF‐PLNG/siRNA scaffold on neuron recovery after therapy, the expression level of tubulin (neuronal differentiation marker), neuron, and neurofilament (macrophage marker), NeuN (neuronal marker), and CD31 (endothelial cells marker; Figure 5). There was more CD68 positive cell signal in the lesion site of GDNF‐PLNG/siMIF and GDNF‐GM groups, while the lesion site of SCI and PLNG/siMIF groups had less tubulin positive cell signals. The findings showed GDNF‐PLNG/siMIF scaffold effectively promoted the earliest phases of neuronal differentiation in the lesion site. In addition, compared with other groups, the fluorescence intensity in the GDNF‐PLNG/siMIF group was stronger, revealing that the GDNF‐PLNG/siMIF scaffold benefited neuron regeneration. Moreover, the number of CD31‐positive cells in the GDNF‐PLNG/siMIF group was increased significantly, indicating that could promote endothelial cell proliferation. In summary, treatment with GDNF‐PLNG/siMIF scaffold exhibited a strong ability to axon regeneration of the remaining neurons and reshape axonal transport at the lesion site with no major organ toxicity (Figure S4).

### 2.7 The molecular mechanism of treatment with GDNF‐PLNG/siMIF scaffold

**FIGURE 6:**
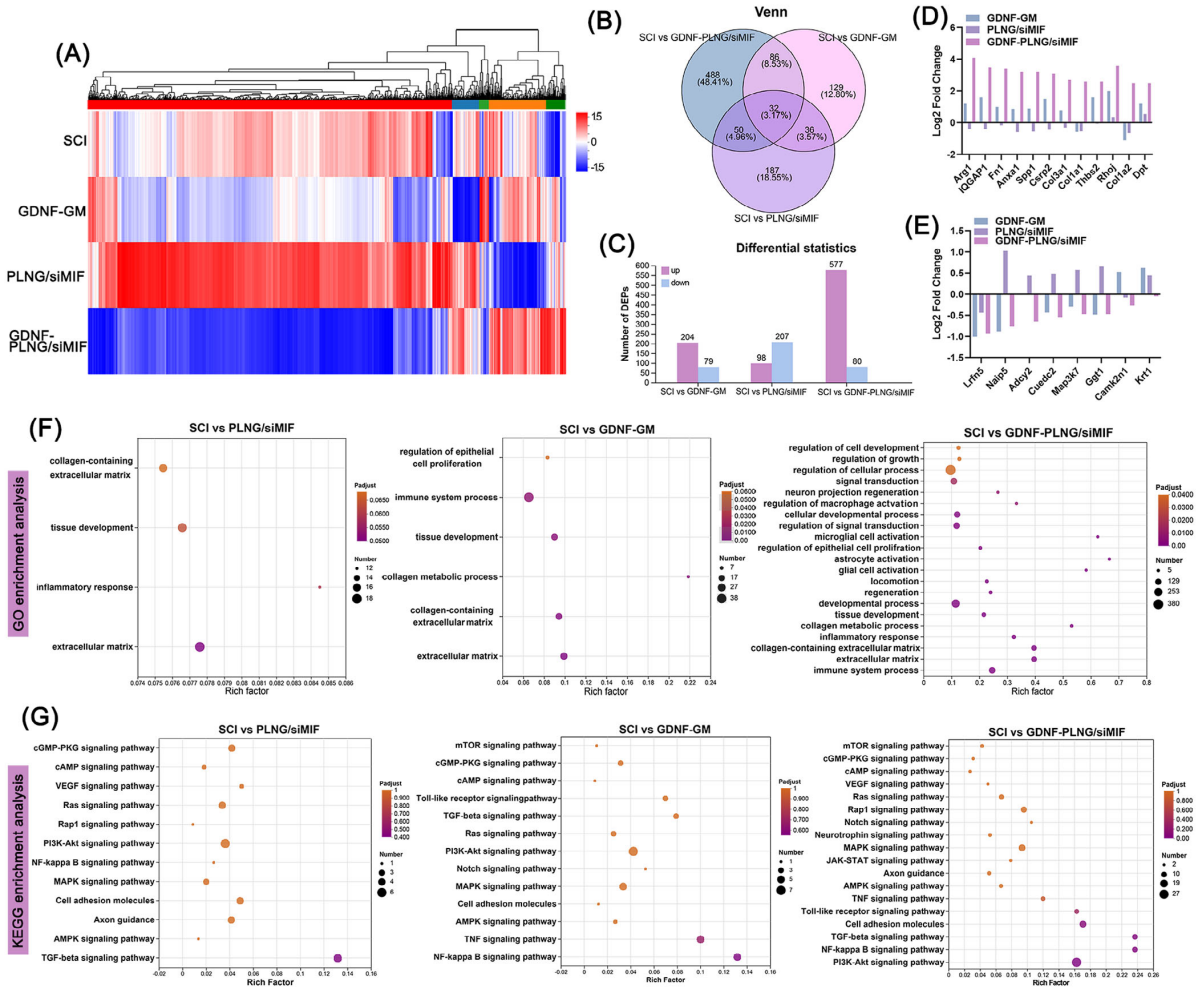
Proteomic analysis of treated with GDNF‐PLNG/siMIF scaffold on regulating inflammatory and immune. (A) The all detected protein expression of each group. (B) Venn diagram of GDNF‐GM, PLNG/siMIF, and GDNF‐PLNG/siMIF groups compared with SCI group. (C) The upregulation and downregulation number of DEPs of each group. (D) Proteins with tissue repair and signal transduction function among the upregulation DEPs. (E) Proteins with inflammation regulation function among the downregulation DEPs. (F) GO enrichment analysis of DEPs in each group. (G) KEGG enrichment analysis of DEPs in each group.

In order to study the interaction mechanism of GDNF protein and MIF siRNA loaded on PLNG scaffold for treatment SCI, the spinal cord tissue of each group underwent proteomics analysis by Astral‐data‐independent acquisition (DIA). All detected protein expressions are shown in Figure 6A, in which plenty of downregulated proteins could be observed in the GDNF‐PLNG/siMIF group. Compared with the SCI group, 656 species of proteins were present in the GDNF‐PLNG/siMIF group, while 283 and 305 species of proteins were present in the GDNF‐GM and PLNG/siMIF groups, respectively (Figure 6B). And only 32 species of proteins were presented in the intersection of GDNF‐GM, PLNG/siMIF, and GDNF‐PLNG/siMIF groups, showing that GDNF‐PLNG/siMIF scaffold inducted abundant protein to participant repairing process. In differentially expressed proteins (DEPs), 577 species of upregulation protein and 80 species of downregulation protein in the GDNF‐PLNG/siMIF group (Figure 6C). These results suggested that the GDNF‐PLNG/siMIF scaffold exhibited the ability to inhibit protein expression and promote protein expression. In all upregulation DEPs, compared with GDNF‐GM and PLNG/siMIF groups, there were many tissue repair and signal transduction functional proteins in the GDNF‐PLNG/siMIF group, such as col3a1, col1a1, col1a2, Fn1, and Anxa1 (Figure 6D). In all downregulation DEPs, many inflammation‐related proteins were found in the GDNF‐PLNG/siMIF group, including Naip5, Adcy2, and Map3k7 (Figure 6E). These indicated that GDNF‐PLNG/siMIF scaffold is involved in promoting the protein expression of tissue repair and also inhibiting inflammatory factors secretion. Moreover, DEPs in each group compared with SCI groups were measured the GO enrichment analysis to identify the main protein functions (Figure 6F). Compared with PLNG/siMIF and GDNF‐GM groups, the kinds of DEPs enrichment in tissue repair and inflammatory regulation were 21 kinds in the GDNF‐PLNG/siMIF group, and DEPs were mostly enriched in regulation of cellular process, developmental process, and signal transduction, which was also enriched on locomotion, microglial, astrocyte, glial, macrophage, and neuron cell regulation. These results demonstrated that treated with GDNF‐PLNG/siMIF scaffold involved in abundant immune response and tissue development. Furthermore, DEPs in each group compared with SCI groups were measured by the KEGG enrichment analysis to identify the specific biological pathway (Figure 6G). Compared with other groups, the kinds of DEPs in GDNF‐PLNG/siMIF group enrichment on the inflammatory regulation pathway involved 18 kinds, which were mostly enriched on PI3K‐Akt, MAPK, and TNF signaling pathways. These results illustrated that GDNF‐PLNG/siMIF scaffold played a key role in the anti‐inflammation pathway and growth factors expression.

**FIGURE 7:**
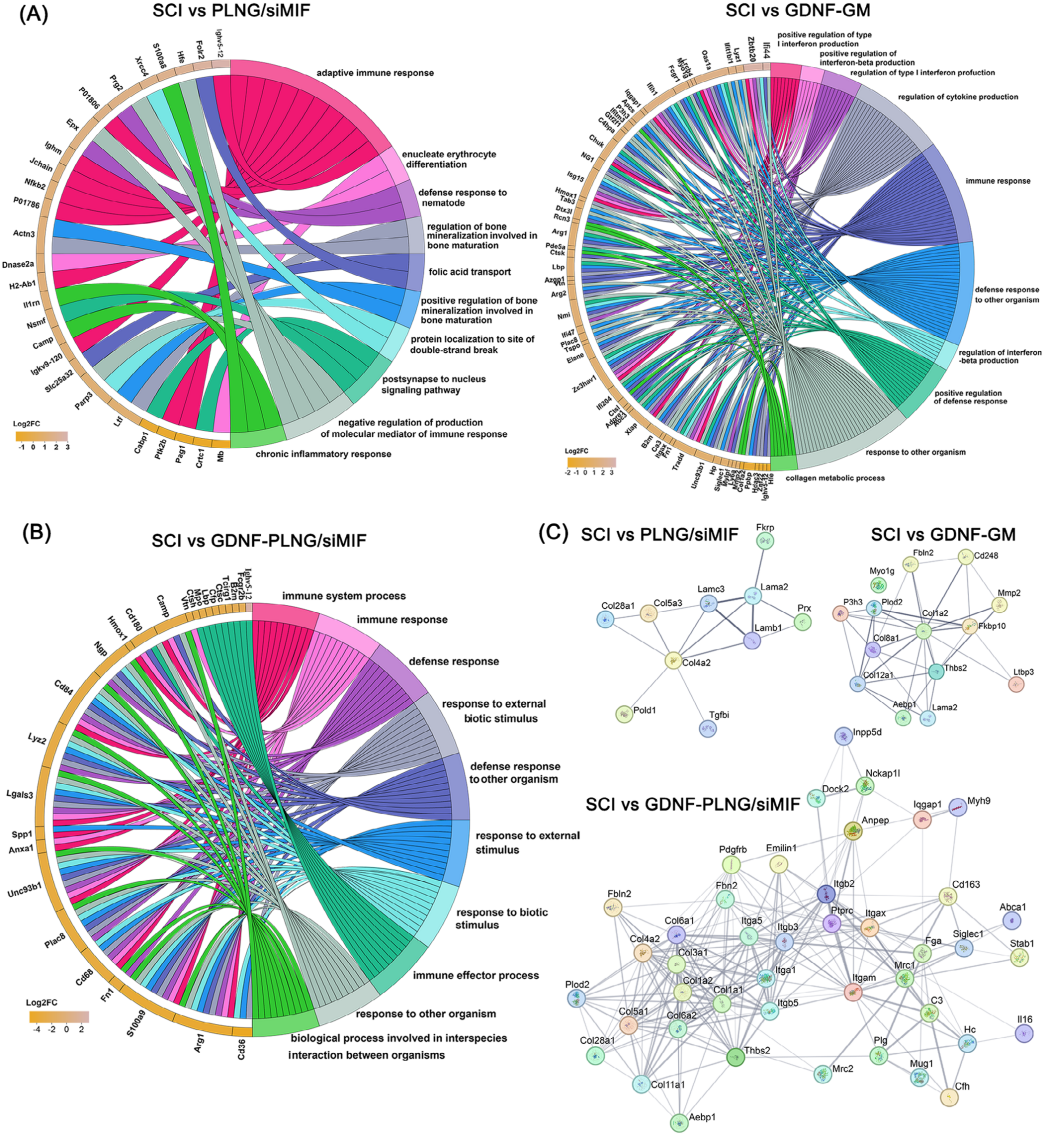
The biological mechanism of treated with GDNF‐PLNG/siMIF scaffold. (A) the top 10 GO terms showing enrichment of DEPs in PLNG/siMIF and GDNF‐GM group, and (B) GDNF‐PLNG/siMIF group. (C) The DEPs of each group constructed a PPI network using the STRING database.

What's more, the top 10 GO terms showing enrichment in DEPs were analyzed to figure out the major biological process of each group. The DEPs in the PLNG/siMIF group were focused on adaptive immune response, while the GDNF‐GM group was focused on immune response and regulation of cytokine production (Figure 7A). In addition, the GDNF‐PLNG/siMIF group was enriched on immune response and biological processes involved in interspecies interaction between organisms, such as signal transduction (Figure 7B). Moreover, the DEPs of each group constructed a protein–protein interaction analysis (PPI) network using the STRING database to figure out the crucial protein in the therapy process (Figure 7C). Compared with PLNG/siMIF and GDNF‐GM groups, the PPI network in the GDNF‐PLNG/siMIF group was more complicated and more protein involved in was interaction. The collagen family protein (Col1a2, Col1a1, Col1a2, Col5a1, Col3a1, Col4a2, Col6a1) and integrins protein (Itga1, Itga5, Itgb3, Itgb5) were found highly interaction in GDNF‐PLNG/siMIF group. Thus, the collagen and integrin proteins were crucial regulation factors for SCI treatment, which could be regarded as therapeutic targets for further research. Therefore, with the interaction mechanism of GDNF protein and MIF siRNA, treatment with GDNF‐PLNG/siMIF scaffold could not only regulate immune and growth factors but also promote the re‐construction of cell signaling and neuron regeneration for SCI therapy.

## 3 DISCUSSION

Our research delved into an injectable, photocurable GDNF‐PLNG/siRNA scaffold system designed for precise delivery of siRNA and co‐delivery of GDNF. This system allows for targeted reduction of neuroinflammation via siRNA targeting the MIF gene, while simultaneously promoting axon regeneration through controlled release of GDNF directly at the lesion site of SCI. Gene therapy strategies have achieved certain therapeutic effects by regulating the lesion environment, offering repair factors, and establishing neuron conduction pathways.^31^, ^32^, ^33^ However, the above therapeutic methods are focused on monotargeted with a slow and limited therapeutic effect. Regarding neuron regeneration and tissue repair, the growth of neuronal cells requires stimulation from neurotrophic factors. Therefore, the combination of protein and siRNA can target different cells and produce diverse therapeutic effects. Researchers have proven that MIF is highly expressed in an injured spinal cord, which could activate inflammatory signaling in astrocytes.^34^, ^35^, ^36^, ^37^, ^38^ In our results, the siMIF/NP complex could turn the M1 phenotype into the M2 phenotype and increase the expression of anti‐inflammatory factors. In addition, the neurotrophic factor of GDNF has been proven to be crucial for neuronal survival, axon growth, and neuroinflammatory.^15^, ^39^, ^40^ We successfully expressed and purified a monomer–dimer mixture of GDNF protein, which could effectively promote neuron axon growth and favor axonal regeneration of neuron. Thus, the combined application of siRNA‐based gene therapy and protein therapy produces a synergistic effect. Compared with the treatment of delivery dual siRNA targeted, one of the targets in this study is to inhibit inflammation with siRNA, while the other is to directly promote nerve repair with neurotrophic factors.^30^ Our research not only exemplifies the combined therapy of siRNA and protein but also demonstrates favorable therapeutic effects targeting different pathways (immunity and neurorepair). Specifically, the protein focuses on neurorepair, while siRNA is targeted at neuroinflammation.

Hydrogel scaffold has the property of biocompatibility and exhibits great potential applied in the tissue engineering field.^41^, ^42^, ^43^ Hydrogel scaffold can be injected into the lesion site of the spinal cord and provide a bridge to the injured spinal cord for tissue repair and neuron regeneration. Compared with these chemical drugs, neurotrophic factors, and stem cells, hydrogel scaffold is required for more demanding conditions for delivering gene complexes, including avoiding damage from the enzyme, loading multiple drugs, controlled‐released, and maintaining the bioactive of gene complex.^44^, ^45^, ^46^ Therefore, some hydrogel scaffolds add various sensitive properties to fit the demanding condition, such as phase transition triggered by temperature, pH, and photo exposure.^47^, ^48^, ^49^ In our study, the GM scaffold was chemically modified with GDNF protein, and mixed with siRNA, NP, and LAP to obtain GDNF‐PLNG/siRNA scaffold, which could be easily photocured with sol–gel transformation when exposed to 405 nm blue light illumination. The combined application of siRNA and protein exemplifies the ability of the PLNG scaffold to maintain drug activity and retain the drugs at the SCI injury site through controlled release, achieving the goal of reducing the frequency of drug administration. Therefore, compared with previous articles, this study exhibits significant differences in terms of drug therapeutic combinations and delivery methods. The innovation lies in the combined use of siRNA and protein therapy, targeting both immunity and neuron regeneration simultaneously, and achieving controlled release for SCI treatment through co‐delivery via a hydrogel scaffold.

**FIGURE 8:**
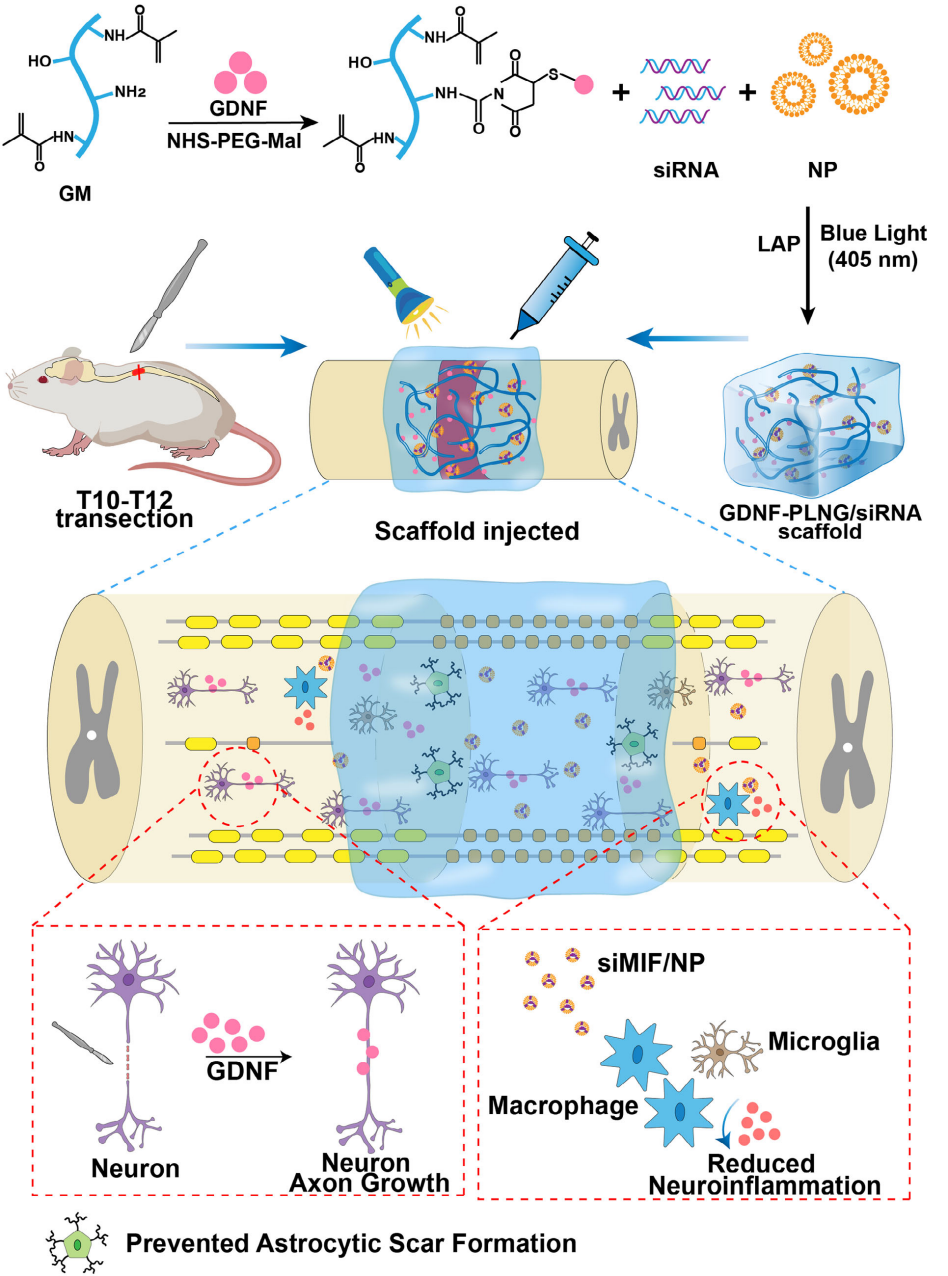
An illustrative overview of the neuron repairing and neuroinflammation reduction treated by the GDNF‐PLNG/siMIF scaffold on the SCI model.

The local administration by hydrogel scaffold can avoid the adverse effects of systematic administration and prevent the drug loss of injection directly to the spinal cord. In our investigation, the formulated GDNF‐PLNG/siRNA scaffold was promptly administered to the injured spinal cord site, encompassing the wound to mitigate bleeding and enhance drug dispersion. As shown in Figure 8, GDNF could effectively promote neuron regeneration and aid in the axonal rejuvenation of neurons. Meanwhile, the siMIF/NP complex could reduce neuron inflammation by promoting macrophage and microglia polarization, hindering the development of astrocytic scars. After being treated with GDNF‐PLNG/siMIF scaffold for three doses, the scaffold exhibited a strong ability to axon regeneration of the remaining neurons and reshape axonal transport at the lesion site. In proteomic analysis, GDNF protein worked in the regulation of cytokine production, while MIF siRNA affected the immune response of anti‐inflammatory. The combined application of these two approaches produces a synergistic effect. In addition, during the therapy process, the collagen and integrin proteins were crucial regulation factors for SCI treatment, which may be regarded as a therapeutic target for further research. In summary, with the interaction of GDNF and MIF siRNA, the GDNF‐PLNG/siMIF scaffold exhibited not only regulated immune and growth factors, but also promoted the re‐construction of cell signaling and neuron regeneration for SCI therapy

## 4 CONCLUSION

In this work, a PLNG scaffold is introduced for efficiently sustained and controlled release of the MIF‐targeted siRNA and co‐delivery of GDNF protein for SCI treatment. The prepared GDNF‐PLNG/siRNA scaffold could maintain the bioactive properties of drugs and control the release to the target site. After local administration of GDNF‐PLNG/siMIF scaffold for SCI, this scaffold could effectively improve the locomotor function of mice and showed a strong ability of tissue repairing and anti‐inflammatory by regulating immune response and cytokine production.

## 5 METHODS

### 5.1 Preparation and characterization of NP and siRNA/NP complex

The synthesis of lipid nanoparticles (NP) was the same as in our previous study. In other words, cationic lipid N‐[1‐(2,3‐dioleoyloxy) propyl]‐N,N,N‐trimethylammonium methyl‐sulfate (DOTAP) and cholesterol were mixed in trichloromethane at a molar ratio of 1:1 . This mixture was completely dissolved under the room temperature, and then the chloroform was removed by rotary evaporation under the temperature of 60°C. After that, a uniform lipid film was obtained and rehydrated with 5% glucose to obtain lipid nanoparticles. This NP was ultrasound (950 W, open 5 s, stop 5 s) for 2 min to increase the disperse of nanoparticles. Simultaneously, siRNA was mixed with NP at a weight ratio of 1:4 at room temperature for 15 min to obtain siRNA/NP complex. The particle size and zeta potential of the NP and siRNA/NP complex were examined by dynamic light scattering (Nano ZS; Malvern Panalytical). The NP and siRNA/NP complex were stained with phosphotungstic acid to observe the morphology by transmission electron microscopy (Tecnai G2 F20 S‐TWIN).

### 5.2 Constructs, expression, and purification of GDNF

The mouse *GDNF* gene (NM_001301357.1) was synthesized by General Biosystems, Inc. The truncated GDNF (113‐211aa) was cloned into a pfastbac1 vector with gp67 signal peptide, 10×His tag, Sumo tag, and TEV protease. GDNF protein was expressed using Sf9 cells with the Bac‐to‐Bac System and was evaluated using western blot analysis with the anti‐His HRP antibody (proteintech, HRP‐66005). For large‐scale expression, Sf9 cells were cultured to a density of 2.5 × 10^6^ and infected with P3 passage Baculovirus for 72 h. The purification protocol was similar to our previous reference (https://doi.org/10.1038/cr.2015.92). In brief, Ni‐affinity beads (Cytiva Life Sciences, 17371201) were added to bind the target protein for 1 h. The column was washed by 20 mM Tris pH 7.4, 150 mM NaCl, and 20 mM imidazole. Subsequently, the protein was eluted with wash buffer with 250 mM imidazole and digested with TEV enzyme at 4°C overnight to remove the His‐Sumo tag. The digested mixtures were separated using a HiTrap Q HP anion exchange chromatography column (Cytiva Life Sciences, 17115401). Further purification was then performed using a Superdex 200 increase 10/300GL column (Cytiva Life Sciences, 28990944) in PBS buffer at pH 7.4. The peak fractions containing the GDNF protein were pooled together, concentrated, and rapidly frozen in liquid nitrogen and stored at −80°C for future use. The FITC‐labelled GDNF was used FITC Quick Labeling Kit (ARL0021K, Frdbio) to prepare for further use.

### 5.3 Synthesis of the GDNF‐PLNG/siRNA scaffold

The synthesis of prepolymer GelMA (GM) solution was the same as our previous study. Briefly, 10 g type A porcine skin gelatin (Sigma‐Aldrich) was melted in 100 mL PBS with stirred magnetically. Then, an 8% mass of methacrylic anhydride (Sigma‐Aldrich) was dropped into the mixture. After the reaction for 3 h, the mixture was dialyzed in deionized water for one week and freeze‐dried to obtain prepolymer GM solution. After that, 1 g of prepolymer GM solution was added to the 0.25% mass of visible‐light sensitive initiator (lithium phenyl‐2,4,6‐trimethylbenzoylphosphinate, LAP, Sigma‐Aldrich) and cross‐linked under blue light with 405 nm wavelength for 30 s to prepare a GM scaffold with a mass concentration of 5%. GDNF protein and Maleimide (polyethylene glycol 2000) NHS (MAL‐PEG_2000_‐NHS, Xi'an ruixi Biological Technology Co., Ltd.) was overnight reacted at 4°C with a molar ratio of 1:1. The mixture was added prepolymer GM solution reacted at 37°C for 8 h and then added LAP solution to obtain GDNF‐GM scaffold (the final concentration of GM solution was 5%). The 5% GDNF‐GM scaffold was mixed up with siRNA, NP, and LAP to prepare a GDNF‐photocurable lipid nanoparticle GelMA loaded siRNA (GDNF‐PLNG/siRNA) scaffold.

### 5.4 The preparation of the SCI model

Seven‐week‐old balb/c mice were used to evaluate the therapy effect of the GDNF‐PLNG/siMIF scaffold. The mice were intraperitoneally injected tribromoethanolto (0.2 mL/10 g) to remain deeply anesthetized. With the T9‐T10 spinous process as the center, an incision was made on the back to remove tissue, spinous process, and lamina to expose the spinal cord. scissors sharp sterile spring was used to transect the spinal cord. Mice were divided into four groups with 10 mice per group. After modeling, 200 µL of 5% GDNF‐GM (GDNF with 35 µg), PLNG/siMIF (siMIF with 35 µg, and NP with 140 µg), and GDNF‐PLNG/siMIF (GDNF with 35 µg, siMIF with 35 µg, and NP with 140 µg) was injected to the injury site and crosslinked immediately, respectively. The drugs were given every 7 days for a total of three times. No treatment was given to the SCI group after successful modeling. During the first administration, the scaffold solution was directly injected into the spinal cord injury site and immediately photocured to the injury site for 30 s; for the second and third administrations, there was no need to open the skin again, and the scaffold solution was directly injected subcutaneously into the spinal cord injury site and then photocured. Antibiotics were applied immediately after surgery in each group. The bladder was squeezed two to four times daily to help urinate until reflex urination was established.

### 5.5 Behavioral evaluations

The BMS and footprint analysis were used to investigate the recovery of motor function in mice after injury and drug administration. The BMS scoring method was used to evaluate the open‐field motor function every other day.

As for footprint analysis, the forelimbs of the mice were stained with red ink and the hindlimbs were stained with blue ink. Underneath the straight track, a sheet of white paper was positioned to enable the mice to walk independently for a minimum of four to five steps. The gait patterns of the mice were captured, and the average values of stride length, stride width, and footprint area were analyzed using the ImageJ software program.

### 5.6 Proteomics analysis

To investigate the interaction of GDNF and MIF siRNA in the SCI repair process, the spinal cord tissue in SCI, GDNF‐GM, PLNG/siMIF, and GDNF‐PLNG/siMIF groups were collected for proteomics analysis. Briefly, the total protein of each group was extracted and digested to obtain peptides. Then, peptide samples were quantified by the NANO DROP ONE (Thermo Scientific) and analyzed by Orbitrap Astral mass spectrometer (Thermo) under the DIA mode. The obtained DIA raw data was searched into NCBI and UniProt database. The chi‐square test was used to calculate the *p*‐values and fold change (FC). Proteins with an FC > 1 and *p* < 0.05 were considered as identified DEPs. The enrichment analysis of DEPs was performed using GO (gene ontology, http://geneontology.org/) and KEGG pathway (Kyoto Encyclopedia of gene and genomes, http://www.genome.jp/kegg/). PPI was performed using the String v11.5.

### 5.7 Statistical analysis

GraphPad Prism 8.0 was used to complete data analysis and drawing, and the experimental results were denoted as mean ± standard deviation. Each group was repeated three times and the statistical methods included the Student's *t*‐test and one‐ or two‐way analysis of variance. The statistical results were considered statistically significant when *p* < 0.05 and significance levels include *p < 0.05, **p < 0.01, ***p < 0.001, and ****p < 0.0001.

## AUTHOR CONTRIBUTIONS

Yan Gao, Kaiyu Wang, and Yi Wu performed writing the manuscript and all the experiment. Jin Zhang and Jingmei Li performed a statistical analysis. Shan Wu and Pingchuan Ma performed animals’ experiment. Guobo Shen and Ke Men conceptualized the study and provided funding acquisition. All authors checked the manuscript and approved it for publication.

## CONFLICT OF INTEREST STATEMENT

The authors declare no conflict of interest.

## ETHICS APPROVAL

The Balb/c mouse was acquired from Beijing HFK Bio‐Technology Co., Ltd. The entire animal experimentation protocol received ethical clearance from the Animal Ethics Committee of Sichuan University's General Administration of Health Research, under approval number 20230310050.

## Supporting information



Supporting Information

## Data Availability

The data are available upon request from the authors.

## References

[1] Krause JS , Cao Y , DiPiro N . Psychological factors and risk of mortality after spinal cord injury. J Spinal Cord Med. 2020;43(5):667‐675. 31794353 10.1080/10790268.2019.1690766 PMC7534322

[2] Griffin JM , Bradke F . Therapeutic repair for spinal cord injury: combinatory approaches to address a multifaceted problem. EMBO Mol Med. 2020;12(3):e11505. 32090481 10.15252/emmm.201911505 PMC7059014

[3] Gao Y , Men K , Pan C , et al. Functionalized DMP‐039 hybrid nanoparticle as a novel mRNA vector for efficient cancer suicide gene therapy. Int J Nanomedicine. 2021;16:5211‐5232. 34366664 10.2147/IJN.S319092 PMC8335320

[4] Lei S , Zhang X , Men K , et al. Efficient colorectal cancer gene therapy with IL‐15 mRNA nanoformulation. Molecular pharmaceutics. 2020;17(9):3378‐3391. 32787272 10.1021/acs.molpharmaceut.0c00451

[5] Lei S , Chen X , Gao Y , et al. ALPPL2‐binding peptide facilitates targeted mrna delivery for efficient hepatocellular carcinoma gene therapy. Advanced Functional Materials. 2022:2204342.

[6] Yang X , Yang W , Xia X , et al. Intranasal delivery of BACE1 siRNA and rapamycin by dual targets modified nanoparticles for Alzheimer's disease therapy. Small. 2022;18(30):e2203182. 35771092 10.1002/smll.202203182

[7] Kandil R , Baldassi D , Bohlen S , et al. Targeted GATA3 knockdown in activated T cells via pulmonary siRNA delivery as novel therapy for allergic asthma. J Control Release. 2023;354:305‐315. 36634709 10.1016/j.jconrel.2023.01.014 PMC7614985

[8] Shan S , Chen J , Sun Y , et al. Functionalized macrophage exosomes with panobinostat and PPM1D‐siRNA for diffuse intrinsic pontine gliomas therapy. Adv Sci (Weinh). 2022;9(21):e2200353. 35585670 10.1002/advs.202200353 PMC9313473

[9] Guo S , Perets N , Betzer O , et al. Intranasal delivery of mesenchymal stem cell derived exosomes loaded with phosphatase and tensin homolog sirna repairs complete spinal cord injury. ACS Nano. 2019;13(9):10015‐10028. 31454225 10.1021/acsnano.9b01892

[10] Rong Y , Wang Z , Tang P , et al. Engineered extracellular vesicles for delivery of siRNA promoting targeted repair of traumatic spinal cord injury. Bioact Mater. 2023;23:328‐342. 36474657 10.1016/j.bioactmat.2022.11.011 PMC9706413

[11] Francos‐Quijorna I , Sanchez‐Petidier M , Burnside ER , et al. Chondroitin sulfate proteoglycans prevent immune cell phenotypic conversion and inflammation resolution via TLR4 in rodent models of spinal cord injury. Nat Commun. 2022;13(1):2933. 35614038 10.1038/s41467-022-30467-5 PMC9133109

[12] Lee D , Baek S , Kim YY , et al. Self‐assembled DNA‐protein hybrid nanospheres: biocompatible nano‐drug‐carriers for targeted cancer therapy. ACS Appl Mater Interfaces. 2022;14(33):37493‐37503. 35969502 10.1021/acsami.2c10397

[13] De Lorenzo F , Luningschror P , Nam J , et al. CDNF rescues motor neurons in models of amyotrophic lateral sclerosis by targeting endoplasmic reticulum stress. Brain. 2023;146(9):3783‐3799. 36928391 10.1093/brain/awad087 PMC10473573

[14] Qian K , Bao X , Li Y , et al. Cholinergic neuron targeting nanosystem delivering hybrid peptide for combinatorial mitochondrial therapy in Alzheimer's disease. ACS Nano. 2022;16(7):11455‐11472. 35839463 10.1021/acsnano.2c05795

[15] Ma J , Li J , Wang X , et al. GDNF‐loaded polydopamine nanoparticles‐based anisotropic scaffolds promote spinal cord repair by modulating inhibitory microenvironment. Adv Healthcare Mater. 2023;12(8):e2202377. 10.1002/adhm.202202377 36549669

[16] Wang L , Zhang D , Ren Y , et al. Injectable hyaluronic acid hydrogel loaded with BMSC and NGF for traumatic brain injury treatment. Mater Today Bio. 2022;13:100201. 10.1016/j.mtbio.2021.100201 PMC8733324 35024600

[17] Zhang F , Wu X , Li Q , et al. Dual growth factor methacrylic alginate microgels combined with chitosan‐based conduits facilitate peripheral nerve repair. Int J Biol Macromol. 2024;268(1):131594. Pt. 38621568 10.1016/j.ijbiomac.2024.131594

[18] Shen H , Xu B , Yang C , et al. A DAMP‐scavenging, IL‐10‐releasing hydrogel promotes neural regeneration and motor function recovery after spinal cord injury. Biomaterials. 2022;280:121279. 34847433 10.1016/j.biomaterials.2021.121279

[19] Jaffer H , Andrabi SS , Petro M , Kuang Y , Steinmetz MP , Labhasetwar V . Catalytic antioxidant nanoparticles mitigate secondary injury progression and promote functional recovery in spinal cord injury model. J Control Release. 2023;364:109‐123. 37866402 10.1016/j.jconrel.2023.10.028 PMC10842504

[20] Feng F , Song X , Tan Z , et al. Cooperative assembly of a designer peptide and silk fibroin into hybrid nanofiber gels for neural regeneration after spinal cord injury. Sci Adv. 2023;9(25):eadg0234. 37352345 10.1126/sciadv.adg0234 PMC10289662

[21] Li J , Yao Y , Wang Y , et al. Modulation of the crosstalk between Schwann cells and macrophages for nerve regeneration: a therapeutic strategy based on a multifunctional tetrahedral framework nucleic acids system. Adv Mater. 2022;34(46):e2202513. 35483031 10.1002/adma.202202513

[22] Chen P , Xu C , Wu P , et al. Wirelessly powered electrical‐stimulation based on biodegradable 3D piezoelectric scaffolds promotes the spinal cord injury repair. ACS Nano. 2022;16(10):16513‐16528. 36174221 10.1021/acsnano.2c05818

[23] Xin W , Baokun Z , Zhiheng C , et al. Biodegradable bilayer hydrogel membranes loaded with bazedoxifene attenuate blood‐spinal cord barrier disruption via the NF‐kappaB pathway after acute spinal cord injury. Acta Biomater. 2023;159:140‐155. 36736849 10.1016/j.actbio.2023.01.056

[24] Zeng X , Wei QS , Ye JC , et al. A biocompatible gelatin sponge scaffold confers robust tissue remodeling after spinal cord injury in a non‐human primate model. Biomaterials. 2023;299:122161. 37236138 10.1016/j.biomaterials.2023.122161

[25] Sha Q , Wang Y , Zhu Z , et al. A hyaluronic acid/silk fibroin/poly‐dopamine‐coated biomimetic hydrogel scaffold with incorporated neurotrophin‐3 for spinal cord injury repair. Acta Biomater. 2023;167:219‐233. 37257575 10.1016/j.actbio.2023.05.044

[26] Gao C , Li Y , Liu X , Huang J , Zhang Z . 3D bioprinted conductive spinal cord biomimetic scaffolds for promoting neuronal differentiation of neural stem cells and repairing of spinal cord injury. Chem Eng J. 2023;451:138788.

[27] Wang R , Wu X , Tian Z , et al. Sustained release of hydrogen sulfide from anisotropic ferrofluid hydrogel for the repair of spinal cord injury. Bioact Mater. 2023;23:118‐128. 36406246 10.1016/j.bioactmat.2022.10.020 PMC9661652

[28] Silva D , Schirmer L , Pinho TS , et al. Sustained release of human adipose tissue stem cell secretome from star‐shaped poly(ethylene glycol) glycosaminoglycan hydrogels promotes motor improvements after complete transection in spinal cord injury rat model. Adv Healthcare Mater. 2023;12(17):e2202803. 10.1002/adhm.202202803 36827964

[29] Liu X , Song S , Chen Z , et al. Release of O‐GlcNAc transferase inhibitor promotes neuronal differentiation of neural stem cells in 3D bioprinted supramolecular hydrogel scaffold for spinal cord injury repair. Acta Biomater. 2022;151:148‐162. 36002129 10.1016/j.actbio.2022.08.031

[30] Gao Y , Wang K , Wu S , et al. Injectable and photocurable gene scaffold facilitates efficient repair of spinal cord injury. ACS Appl Mater Interface. 2024;16(4):4375‐4394. 10.1021/acsami.3c14902 38185858

[31] Zhang J , Li Y , Xiong J , et al. Delivery of pOXR1 through an injectable liposomal nanoparticle enhances spinal cord injury regeneration by alleviating oxidative stress. Bioact Mater. 2021;6(10):3177‐3191. 33778197 10.1016/j.bioactmat.2021.03.001 PMC7970014

[32] Song Z , Wang Z , Shen J , Xu S , Hu Z , Nerve growth factor delivery by ultrasound‐mediated nanobubble destruction as a treatment for acute spinal cord injury in rats. Int J Nanomedicine. 2017;12:1717‐1729. 28280337 10.2147/IJN.S128848 PMC5340249

[33] Leibinger M , Zeitler C , Gobrecht P , Andreadaki A , Gisselmann G , Fischer D . Transneuronal delivery of hyper‐interleukin‐6 enables functional recovery after severe spinal cord injury in mice. Nat Commun. 2021;12(1):391. 33452250 10.1038/s41467-020-20112-4 PMC7810685

[34] Bavencoffe AG , Spence EA , Zhu MY , et al. Macrophage migration inhibitory factor (MIF) makes complex contributions to pain‐related hyperactivity of nociceptors after spinal cord injury. J Neurosci. 2022;42(27):5463‐5480. 35610050 10.1523/JNEUROSCI.1133-21.2022 PMC9270921

[35] Zhang H , Hu YM , Wang YJ , et al. Macrophage migration inhibitory factor facilitates astrocytic production of the CCL2 chemokine following spinal cord injury. Neural Regen Res. 2023;18(8):1802‐1808. 36751809 10.4103/1673-5374.363184 PMC10154479

[36] Piri SM , Ghodsi Z , Shool S , et al. Macrophage migration inhibitory factor as a therapeutic target after traumatic spinal cord injury: a systematic review. Eur Spine J. 2021;30(6):1474‐1494. 33486594 10.1007/s00586-021-06718-2

[37] Zhang Y , Zhou Y , Chen S , et al. Macrophage migration inhibitory factor facilitates prostaglandin E2 production of astrocytes to tune inflammatory milieu following spinal cord injury. J Neuroinflammation. 2019;16(1):85. 30981278 10.1186/s12974-019-1468-6 PMC6461812

[38] Zhou Y , Guo W , Zhu Z , et al. Macrophage migration inhibitory factor facilitates production of CCL5 in astrocytes following rat spinal cord injury. J Neuroinflammation. 2018;15(1):253. 30180853 10.1186/s12974-018-1297-z PMC6122456

[39] Baloh RH , Johnson JP , Avalos P , et al. Transplantation of human neural progenitor cells secreting GDNF into the spinal cord of patients with ALS: a phase 1/2a trial. Nat Med. 2022;28(9):1813‐1822. 36064599 10.1038/s41591-022-01956-3 PMC9499868

[40] Hunt CPJ , Penna V , Gantner CW , et al. Tissue programmed hydrogels functionalized with GDNF improve human neural grafts in Parkinson's disease. Adv Funct Mater. 2021;31(47)

[41] Zheng D , Wang K , Bai B , Hu N , Wang H . Swelling and glyphosate‐controlled release behavior of multi‐responsive alginate‐g‐P(NIPAm‐co‐NDEAm)‐based hydrogel. Carbohydr Polym. 2022;282:119113. 35123748 10.1016/j.carbpol.2022.119113

[42] Teal CJ , Hettiaratchi MH , Ho MT , et al. Directed evolution enables simultaneous controlled release of multiple therapeutic proteins from biopolymer‐based hydrogels. Adv Mater. 2022;34(34):e2202612. 35790035 10.1002/adma.202202612

[43] Phan VHG , Murugesan M , Huong H , et al. Cellulose nanocrystals‐incorporated thermosensitive hydrogel for controlled release, 3D printing, and breast cancer treatment applications. ACS Appl Mater Interfaces. 2022;14(38):42812‐42826. 36112403 10.1021/acsami.2c05864

[44] Nazemi Z , Nourbakhsh MS , Kiani S , et al. Co‐delivery of minocycline and paclitaxel from injectable hydrogel for treatment of spinal cord injury. J Control Release. 2020;321:145‐158. 32035190 10.1016/j.jconrel.2020.02.009

[45] Boido M , Ghibaudi M , Gentile P , Favaro E , Fusaro R , Tonda‐Turo C . Chitosan‐based hydrogel to support the paracrine activity of mesenchymal stem cells in spinal cord injury treatment. Sci Rep. 2019;9(1):6402. 31024032 10.1038/s41598-019-42848-w PMC6483991

[46] Zhao YZ , Jiang X , Lin Q , et al. Thermosensitive heparin‐poloxamer hydrogels enhance the effects of GDNF on neuronal circuit remodeling and neuroprotection after spinal cord injury. J Biomed Mater Res A. 2017;105(10):2816‐2829. 28593744 10.1002/jbm.a.36134

[47] Long Y , Yan L , Dai H , et al. Enhanced proliferation and differentiation of neural stem cells by peptide‐containing temperature‐sensitive hydrogel scaffold. Mater Sci Eng C Mater Biol Appl. 2020;116:111258. 32806302 10.1016/j.msec.2020.111258

[48] Han Z , Wang P , Mao G , et al. Dual pH‐responsive hydrogel actuator for lipophilic drug delivery. ACS Appl Mater Interfaces. 2020;12(10):12010‐12017. 32053341 10.1021/acsami.9b21713

[49] Nguyen HT , Massino M , Keita C , Salmon JB . Microfluidic dialysis using photo‐patterned hydrogel membranes in PDMS chips. Lab Chip. 2020;20(13):2383‐2393. 32510526 10.1039/d0lc00279h

