# Supplementary material for: Controlled release of MIF siRNA and GDNF protein from a photocurable scaffold efficiently repairs spinal cord injury
Source: MedComm (2020). 2025 Feb 17;6(3):e70099. doi: 10.1002/mco2.70099 (PMC11831192; doi:10.1002/mco2.70099)
Supplement: Supplementary file 1 — Supporting Information [file MCO2-6-e70099-s001.docx]

**Supporting Information**

**Controlled-release of MIF siRNA and GDNF Protein from a Photocurable Scaffold Efficiently Repairs Spinal Cord Injury**

Yan Gao^1, #^, Kaiyu Wang^1,^ ^#^, Yi Wu^1,^ ^#^, Shan Wu^1^, Pingchuan Ma^2^, Jin Zhang^1^, Jingmei Li^1^, Guobo Shen^1, *^, Ke Men^1, *^

^1^ Department of Biotherapy, Cancer Center and State Key Laboratory of Biotherapy, West China Hospital, Sichuan University, Chengdu ,610041, China.

^2^ State Key Laboratory of Oral Diseases & National Center for Stomatology & National Clinical Research Center for Oral Diseases & Department of Head and Neck Oncology, West China Hospital of Stomatology, Sichuan University, Chengdu 610041 Sichuan, China;

**Correspondence:**

**GuoBo Shen**

State Key Laboratory of Biotherapy and Cancer Center, West China Hospital of Sichuan University, Chengdu 610041, People’s Republic of China

Email shenguobo@126.com

**Ke Men**

State Key Laboratory of Biotherapy and Cancer Center, West China Hospital of Sichuan University, Chengdu 610041, People’s Republic of China

Email mendingbob@hotmail.com

^#^These authors contributed equally to this work.

1. **Materials and Methods**
   1. **Materials**

human embryonic kidney (HEK) cell line 293T (database matching degree 87.5%) and macrophage cell line RAW264.7 (database matching degree 97.56%) were purchased from American Tissue Culture Collection (ATCC) with Short tandem repeat (STR) analysis. Hippocampal neuronal cell line HT22 were purchased from Procell (China, Wuhan) with STR identification (database matching degree 96.77%). Cells were cultured in Dulbecco’s modified Eagle’s medium (DMEM) with penicillin (100 U/mL), streptomycin (100 U/mL), and 10% fetal bovine serums (cell-box, aoke biotechnology). Spodoptera frugiperda insect cells (Sf9 cell line, Thermo Fisher Scientific, 11496015) are used for packaging baculovirus and expressing the GDNF protein. These cells are cultured in serum-free SIM-SF medium (Sino Biological Inc., MSF1). The cells are maintained in a constant-temperature shaking incubator at 27℃ and 120 rpm/min. The murine macrophage migration inhibitory factor small interfering RNA (siMIF) (sense 5’-CCGCAACUACAGUAAGCUG-3’, antisense 5’-CAGCUUACUGUAGUUGCGG-3’), negative control scramble siRNA (scr) (sense 5’-UUCUCCGAACGUGUCACGUTT-3’, antisense 5’-ACGUGACACGUUCGGAGAATT-3’), and siRNA fluorescently labeled with Cy3-siRNA were synthesized by GenePharma (shanghai, China).

- 1. **Scanning electron microscope (SEM) analysis**

To observe the surface morphology of GM scaffold, PLNG scaffold, and GDNF-PLNG/siMIF scaffold, the SEM analysis was tested. The samples were completely freeze at -80°C and the freeze-dried samples were sliced to 2-3 mm and sprayed with gold. The surface characteristics of scaffolds were observed by EVO10 SEM system (Zeiss, Oberkochen, Germany).

- 1. **X-ray photoelectron spectroscopy (XPS) analysis**

To evaluate the chemical modification of GDNF protein, the surface element content of GM scaffold, GDNF protein, and GDNF-GM scaffold were compared by XPS. Briefly, the samples were added to 3 × 3 mm glass slides, dried at 37 ℃, and then detected by AXIS Supra (Kratos).

- 1. **Rheological Properties**

The rotational rheometer (Haake Mars 40) was used to evaluate the rheological properties of GM and GDNF-PLNG/siMIF scaffold. The samples were put on the parallel plate and tested with the shear rate sweep of 0-200 s^-1^ to examine the viscosity of GM and GDNF-PLNG/siMIF scaffold.

- 1. **FT-IR spectroscopy**

To evaluate the chemical structures of GM and GDNF-PLNG/siMIF scaffold, the FT-IR spectroscopy was tested. The samples were prepared and completely freeze at -80°C, then the freeze-dried samples were grinded into powder. The detection method was ATR detection mode with a resolution of 4 cm^-1^ and 16 scans. The wavenumber in the range of 400 to 4000 cm^-1^ was scanned to obtain the infrared absorption spectrum.

- 1. **Degradation character of scaffold *in vitro* and *in vivo***

To assess the in vitro degradation properties, the 5% PLNG scaffold was labeled with blue fluorescent dyes through an Alkene coupling hydrogel process (EFL), then added 100 μL of stained PLNG scaffold to 48-well to form a round and crosslinked under the blue light of 405 nm. The different mass of collagenase IV was added and put in 37 ℃ for 24 h. Then, the supernatant of each well was harvested to determine the fluorescence intensity by Synergy™ HTX Multi-Mode Microplate Reader (BioTek) and the undegraded scaffold was imaged by Chemiluminescence Imaging Systems (Bio-Rad).

As for in the body, 150 μL of 5% GM scaffold or GDNF-PLNG/siMIF scaffold (siMIF, 5μg) was administered in mice subcutaneously. After crosslinked under the blue light of 405 nm, the scaffold formed a bulge under the mouse skin and used the formulation of 0.5 × length × width^2^ to record the volume for every day. On the day 0 and 7, the mouse was sacrificed and cut the skin containing the scaffold for photography.

- 1. **The morphology of NPs in sacffold**

To observe the existence of NPs after scaffold degradation, PLNG scaffold was prepared and digested by 1 mg/mL collagenase IV. The supernatant was collected and tested by dynamic light scattering and TEM.

- 1. **Cytotoxicity assay**

The 3-(4,5-dimethylthiazol-2-yl)-2,5-diphenyltetrazolium bromide (MTT) assay was conduct to evaluate the cytotoxicity of NP *in vitro*. Firstly, 293T cell lines and RAW264.7 cell lines was plated in 96-well plate with 5×10^3^ cells per well. After incubated for 24 hours, the culture was removed and treated with different concentration of NP. In addition, Polyethyleneimine (Mw=25,000, PEI25K, Sigma) was used to be a standard control material. After transfected for 48 hours, 20 μL of the MTT was put into each well with incubation of 4 hours at 37 ℃, and then added 150 μL of DMSO to dissolve the precipitation. After the precipitate in the plate was completely dissolved, the Spectramax Absorbance Reader (Molecular Devices, USA) was used to detect the absorbance at 570 nm.

- 1. **Gel retarding assay**

To detect the binding ability of NP with siRNA, the different ratio of NP was used to incubate with siRNA. Briefly, siMIF (0.5 μg) added to different ratio of NP to form a complex. The samples were electrophoresed on 1% (w/v) agarose gel afor 15 minutes at 120 V. The agarose gel was imaged by E-gel imager (BioRad) before stained with GoldView II Nuclear Staining Dyes (Solarbio).

- 1. **RNase protection assay**

The siRNA/NP complex was treated with RNase A (Solarbio) to study the anti-degradation ability of NP for protecting siRNA from RNase degradation in vitro. Firstly, the final concentration in 0.25 mg/mL of RNase A was added to naked siRNA or siRNA (0.5 μg) /NP complex and incubated at 37 ℃ with different time point (0, 0.25, 1, 2, 4 h). In addition, the siRNA (0.5 μg) /NP complex was incubated with RNase A for 1, 3, 5, 7 days to evaluate a longer anti-degradation capability of NP in vitro. After that, the sodium dodecyl sulfate (1 mg/mL) was used to release the siRNA by incubated with siRNA/NP complex for 10 min in 70 ℃ water bath. All samples were examined by electrophoresis and imaged.

- 1. ***In vitro* Transfection of NP**

The RAW264.7 and HT22 cell lines were treated with the Cy3-siRNA/NP complex in order to evaluate the transfection capability of NP. Specifically, 1×10^5^ RAW264.7 or HT22 cells were seeded into each well of a 24-well plate and allowed to grow for 24 hours. Following this incubation period, the cells were exposed to the Cy3-siRNA (1 μg)/NP complex for an additional 24 hours. Subsequently, the transfection efficiency of the NP was quantified using flow cytometry (NovoCyte Flow Cytometer).

- 1. **Quantitative real-time PCR (Q-PCR)**

The Q-PCR analysis was measured to detect the silence effect of gene complex. RAW264.7 cells were plated in 6-well plate with a density of 4×10^6^ cells per well. After 24 hours of culturing, the scr (4 μg)/NP or siMIF (4 μg)/NP complexes were introduced into the plate. Total RNA was then extracted from the cells using the FastPure® Cell/Tissue Total RNA Isolation Kit V2 (Vazyme). This RNA was subsequently reverse transcribed into cDNA templates utilizing a reverse transcription kit from Vazyme. The relative difference multiple of MIF mRNA level of each group was calculated with 2^-ΔΔCT^ method. The PCR amplification employed primers specific for MIF (forward: 5’-GCCAGAGGGGTTTCTGTCG-3’, reverse 5’-GTTCGTGCCGCTAAAAGTCA-3’) and β-actin (forward: 5’-CCCAGGCATTGCTGACAGG, reverse: 5’-TGGAAGGTGGACAGTGAGGC).

- 1. **Axonal outgrowth of Primary Spinal Cord Neuron stimulated by GDNF**

Spinal cord neurons from a 3-day-old Balb/c mouse were dissected and isolated as primary cells. The neonatal mice were euthanized in chamber with isoflurane and spray with 75% ethanal. The spine of mice was removed, picked out the spinal cord tissue, cut the spinal cord into 1 mm^3^ fragments, and put them into HBSS containing penicillin/streptomycin. After washing the tissue once with HBSS, these fragments were digested with trypsin at 37℃ for 10 min. Then added FBS to terminate the trypsin reaction. Next, using 1 mL pipette tip aspirated the fragments up and down gently to obtain single cells. These supernatants were filter with 80 and 40 μm cell strainers. The filtrate was centrifuged at 300g for 5 minutes, and the supernatant is discarded; the precipitate was re-suspended with mouse spinal cord neuron cell complete medium (Procell, CM-M178). 1 × 10^5^ primary spinal cord neurons were plated into chamber slides (Millipore) re-coated with Poly-L-Lysine (PLL, procell).

In order to evaluate the function of GDNF protein, GDNF (5μg) was added in each chamber. The axonal outgrowth of the primary spinal cord neurons was recorded at 0, 24, and 48 h. After incubation for 48 h, the neuron cells were fixed with paraformaldehyde, permeabilized by 0.1% Triton X-100, blocked with FBS. Then the neuron cells were stained with anti-160 kD neurofilament medium antibody (NF-160, 1:50, ab254348; Abcam). After incubation for 4°C overnight, the neuron cells were stained with goat anti-rabbit Alexa 488 (1:1,000, ab150077; Abcam) and Hoechst for 1 h. The primary spinal cord neurons were observed by fluorescence microscopy (Imager Z2, Zeiss).

- 1. **Controlled release of PLNG scaffold *in vitro***

In order to evaluate the protein and gene complex released from PLNG scaffold, 70 μL of 5% PLNG scaffold loaded with FITC-labelled GDNF (5 μg) and Cy3-siRNA (5 μg) and added to 24-well plate form a round. After crosslinked, scaffold was treated with collagenase IV (1 μg) in PBS at 37 °C. The drug release of protein and siRNA from scaffold was imaged by fluorescence microscopy at 0, 8, 24, 36,72, and 78 h.

- 1. **GDNF release from GM scaffold**

To assess the release of protein drugs from the scaffold, 500 μL of GDNF (7 μg)-GM scaffold was crosslinked in each well of a 24-well plate. Subsequently, 500 μL of PBS was added to the scaffold, and the supernatant was collected. This process was repeated daily for 7 days, with fresh 500 μL of PBS being added each time. The collected supernatants were then analyzed using a BCA assay kit (Thermo) to detect and quantify the released proteins.

- 1. **siRNA release from PLNG scaffold**

To evaluate the siRNA released from scaffold, 500 μL of siMIF (1 μg)/PLNG scaffold was crosslinked in 24 well plate. Then, 500 μL of PBS was put on the scaffold. The scaffold in well was degraded by collagenase and the supernatant was collected every day for a total of 7 days. As control, the siMIF (1 μg)/NP complex was added to 24-well plate with 500 μL PBS. The gene complex was left untreated in the well and the supernatant was collected every day for a total of 7 days. The collected supernatants were detected and analyzed by the Quant-iT^TM^ RiboGreen RNA assay kit (R11490, thermo), and “low-range assay” was adopted as the standard curve.

- 1. **The transfection ability and Knockdown of released nanoparticles**

To assess the transfection potential of the released siRNA/NP complex, 500 μL of Cy3-siRNA (1 μg)/PLNG scaffold was crosslinked in 24-well plates. Following this, 500 μL of serum-free DMEM was added to the scaffold. Over the course of 7 days, the scaffold in each well was degraded using collagenase, and the supernatant containing the released siRNA/NP complex was collected daily. This supernatant was then added to RAW264.7 cells, which were subsequently stained with Hoechst after 24 hours and observed under a fluorescence microscope (Imager Z2, Zeiss) for visualization. Additionally, flow cytometry was employed to quantify the transfection efficiency of the released siRNA/NP complex.

To evaluate the gene-silencing capability of the released siRNA/NP complex, the supernatants containing the complex were harvested daily from the scaffold over 7 days, as described above, and introduced into RAW264.7 cells for 24 hours of incubation. Subsequently, the total RNA was extracted from these cells to analyze the MIF mRNA levels through Q-PCR, thereby assessing the extent of gene silencing achieved by the released siRNA/NP complex.

- 1. **The anti-inflammation mechanism of siMIF/NP complex**

In order to verify the anti-inflammation effect of MIF siRNA *in vitro*, the LPS-stimulation assay and the expression levels of inflammatory factors were detected. Briefly, RAW264.7 cells were seeded in 6-well plate with a density of 5 × 10^4^ and added LPS (at a final concentration with 5 μg/mL) with free-serum DMEM culture. After stimulated for 12 h, the free-serum DMEM culture was change to DMEM with serum and transfected with siMIF (2 μg)/NP complex for 24 h. The RAW264.7 cells were collected and stained with F4/80 (PE) and CD86 (PerCP). Then, the cells were fixation and permeabilization before staining with CD206 (FITC). The M1 macrophage (F4/80^+^ CD86^+^) and M2 macrophage (F4/80^+^ CD206^+^) was detected by flow cytometry. In addition, the total RNA of treated RAW264.7 cells was also extracted and detected the inflammation factors level in cell, including TNF-α (forward: 5’- CAGGCGGTGCCTATGTCTC-3’, reverse 5’-CGATCACCCCGAAGTTCAGTAG-3’), IL1β (forward: 5’-GCAACTGTTCCTGAACTCAACT-3’, reverse 5’- ATCTTTTGGGGTCCGTCAACT-3’), IL10 (forward: 5’- GCTCTTACTGACTGGCATGAG-3’, reverse 5’-CGCAGCTCTAGGAGCATGTG-3’), and IL4 (forward: 5’-GGTCTCAACCCCCAGCTAGT-3’, reverse 5’- GCCGATGATCTCTCTCAAGTGAT-3’)

- 1. **Histological and Immunofluorescent Analysis**

To explore the extent and mechanism of spinal cord recovery, the spinal cord of each groups was collected and stained. Briefly, the mice were intraperitoneally injected with tribromoethanolto (0.2mL/10g). After the mice were deeply anesthetized, the chest cavity was opened and the heart was perfused with PBS. The back muscles and spine of the mice were removed, and the complete spinal cord tissue of each group was collected for photography. Then, the spinal cord was exposed with the injured spinal cord as the center, and the spinal cord tissue of about 1 cm in length was cut. After fixation and dehydration, it was embedded in OCT and stored in -80 ℃ for further use. The spinal cord of each groups was frozen sectioned into a 10 μm slices. The slices were permeabilized with 0.1% Triton X-100 and blocked with 5% goat serum. After that, slices the following antibodies was added on the slices and incubated at 4°C overnight: CD68 (ab125212, abcam), glial fibrillary acidic protein (GFAP, ab68428, Abcam), ionized calcium binding adaptor molecule 1 (IBA1, GT10312, Thermo), CD31 (ab28364, Abcam), beta-3 tubulin (tubulin, MA1-118, Thermo), and neuron (ab104224, Abcam). After three times wash in PBS, slices were covered with the following secondary antibody for 1 h: goat anti-rabbit conjugated with Alexa Fluor 488 (ab150077, Abcam) and goat anti-mouse conjugated with Alexa Fluor 594 (ab150116, Abcam). After that, sections were stained with DAPI (S36964, Thermo), slid coverslip, and imaged by fluorescence microscope (Imager Z2, Zeiss). In addition, the spinal cord slices and the major organs of each groups were treated with hematoxylin and eosin (H&E) staining and the recovery degree of nerve tissue inspinal cord slices were investigated by dying with Luxol Fast Blue Stain Kit (ab150675, abcam).


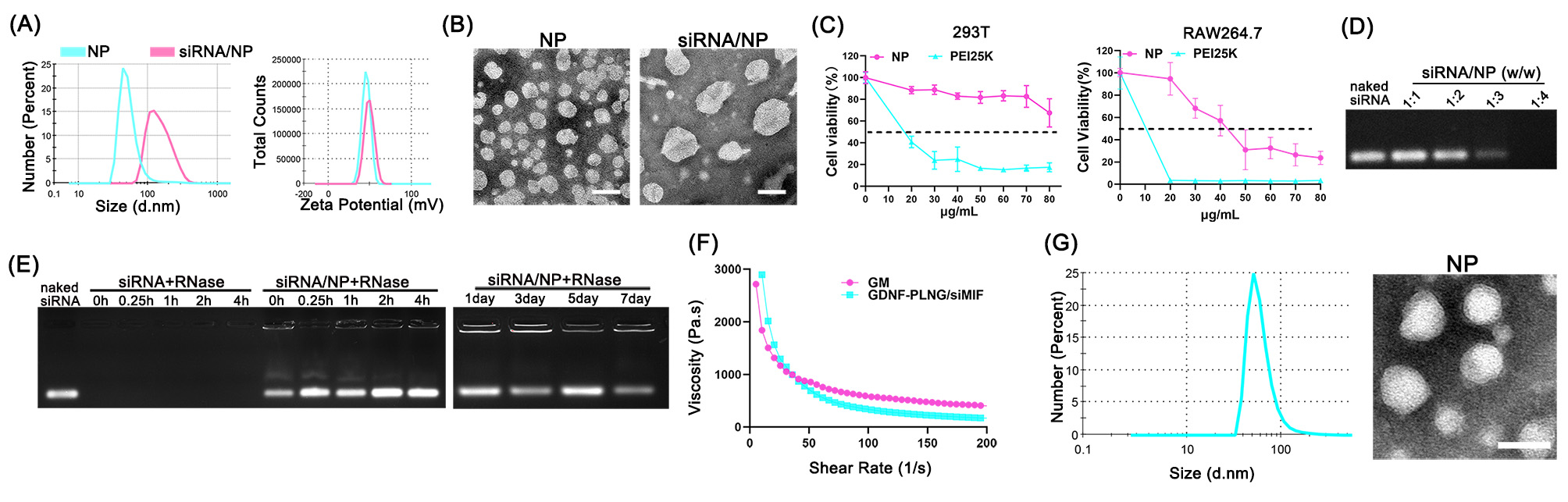


Figure S1: (A) the mean particle size and zeta potential of NP as well as siRNA/NP complex. (B) the morphology of NP and siRNA/NP complex observed by TEM (scale bar, 100 nm). (C) the toxicity of NP and PEI25K tested on 293T and RAW264.7 cells. (D) the binding ratio of siRNA/NP complex. (E) the RNase protection ability of siRNA/NP complex at 0, 0.25, 1, 2, 4 h and even at 1, 3, 5, 7 days. (F) the flow properties and viscosity of GM scaffold and GDNF-PLNG/siMIF scaffold. (G) the mean particle size and morphology of NP degraded from scaffold.


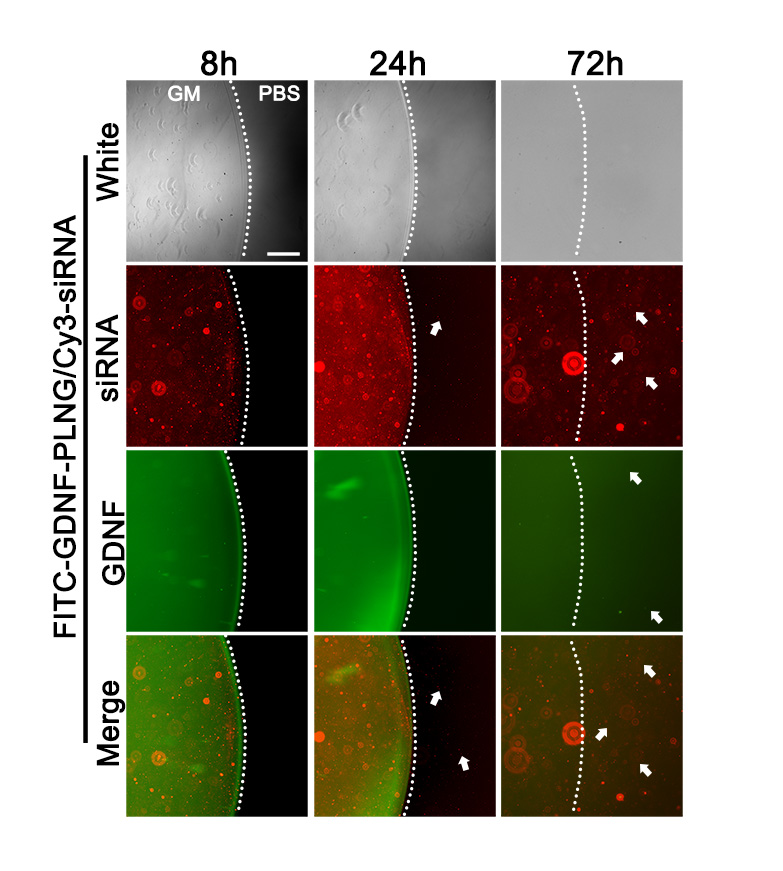


Figure S2: the release and degradation process of FITC-GDNF-PLNG/Cy3-siRNA scaffold (scale bar, 400 μm).


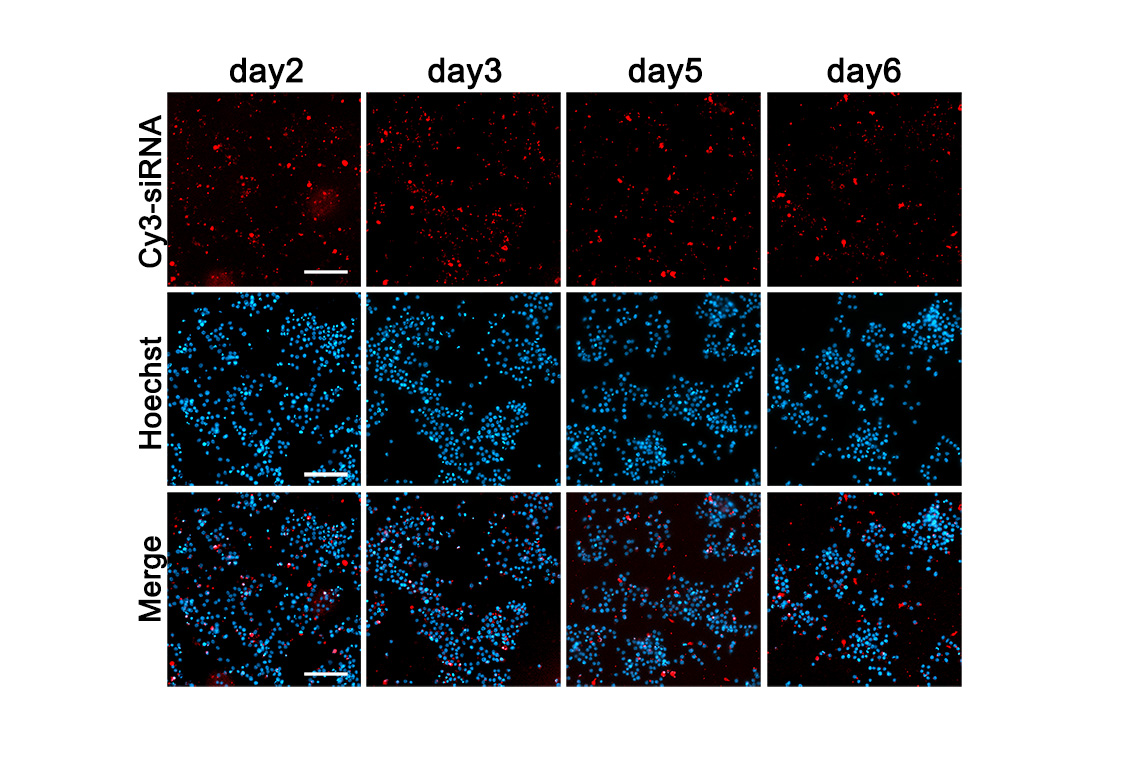


Figure S3: the release rate of GDNF from GM scaffold for seven days. (O) the amount of siRNA released from PLNG scaffold over a period of seven days.


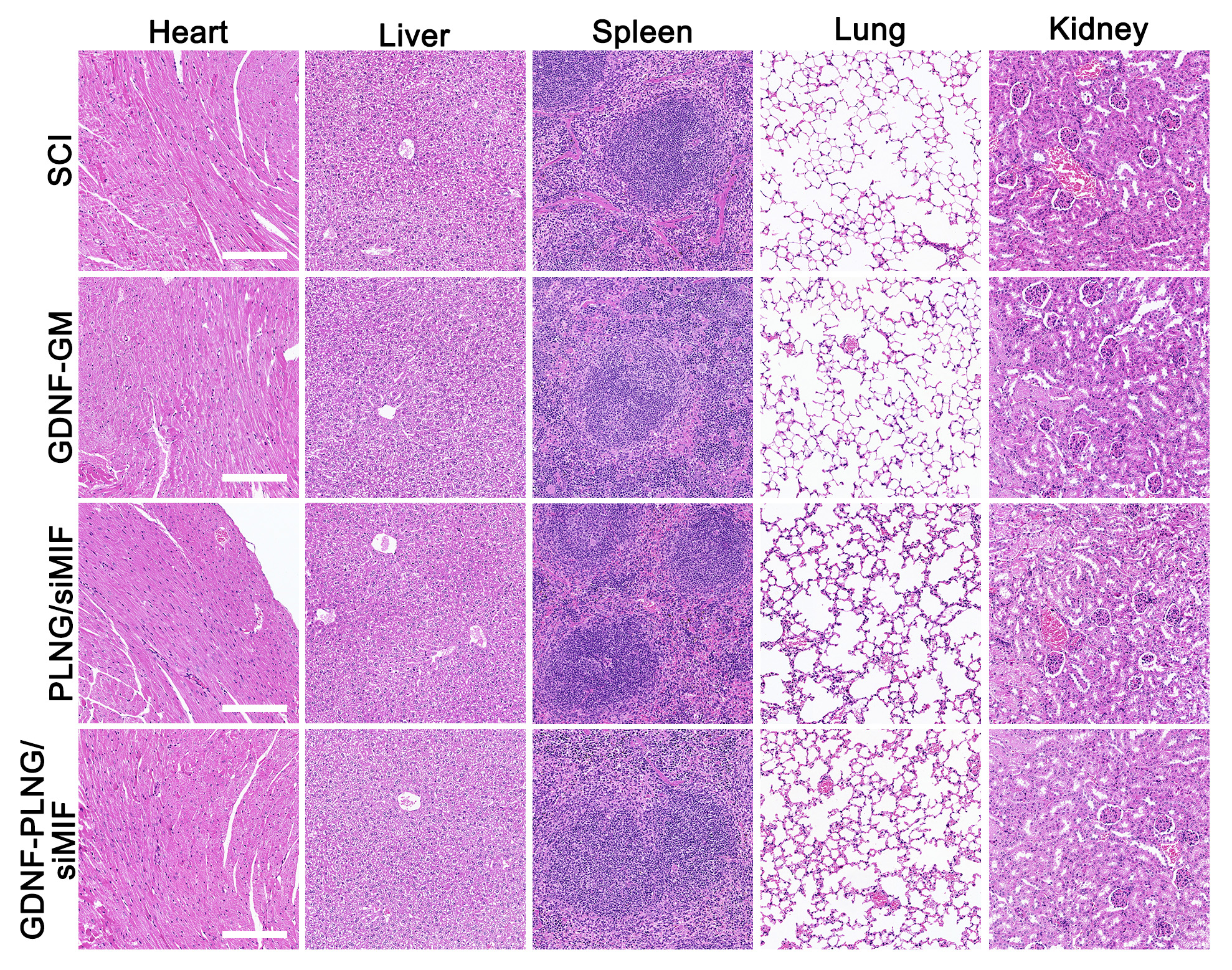


Figure S4: H&E staining of organs after treatment with GDNF-PLNG/siMIF scaffold.
